# Heavy-tailed connections support robust neural communication

**DOI:** 10.64898/2026.10.21.760834

**Published:** 2026-10-26

**Authors:** Jose M. Betancourt, Varun Varanasi, Damon A. Clark, Christopher W. Lynn

**Affiliations:** 1 Department of Physics, Yale University, New Haven, CT 06520, USA; 2 Quantitative Biology Institute, Yale University, New Haven, CT 06520, USA; 3 Harvard Graduate Program in Biophysics, Harvard University, Cambridge, MA 02139, USA; 4 Department of Molecular Cellular and Developmental Biology, Yale University, New Haven, CT 06520, USA; 5 Department of Neuroscience, Yale University, New Haven, CT 06520, USA; 6 Wu Tsai Institute, Yale University, New Haven, CT 06520, USA

## Abstract

In the synaptic wiring between neurons, some rare connections are orders of magnitude stronger than most. This heavy-tailed connectivity is ubiquitous across different species, brain regions, and cell types, suggesting that it serves a general purpose for neural function. Here, we show, both analytically and in simulations, that heavy-tailed connections reduce communication errors in the presence of synaptic noise. In minimal models of individual neurons, noisy connections induce errors at a rate that depends only on a simple network statistic, which we refer to as robustness. For a fixed number of incoming synapses, neurons with broader distributions of connection strength are more robust and, therefore, admit fewer errors. Across several connectomes, we find that real neurons are consistently more robust than comparable null networks. In the whole brain of the fruit fly *Drosophila*, robustness increases with distance from the sensory periphery, such that central neurons are more robust than those involved in sensory processing. Together, these results indicate that heavy-tailed connections may arise universally to fortify the brain against noise in communication.

Neurons communicate through physical connections composed of synapses.^1^ Competing with many sources of biological noise, signals propagate along synaptic wiring to execute vital neural functions.^2^ The revolution in connectomics now provides maps of these connections across multiple species, from *C. elegans*,^3, 4^ mice,^5^ and zebrafish^6^ to the entire brain of the fruit fly *Drosophila*.^7-10^ Universally, these networks share a defining feature: connection strengths are heavy-tailed.^11, 12^ While most neurons interact only weakly, a handful of important connections are orders of magnitude stronger, forming the backbone of neural circuitry. Yet despite this ubiquity, the advantage of heavy-tailed connections for neural function remains unclear.

Take the complete adult brain of the female fruit fly, which consists of nearly 140,000 neurons, each with an average of 109 incoming connections (Table 1).^7, 8^ In invertebrates like the fly, connections typically grow stronger by increasing the number of synapses, such that synapse count approximates functional connection strength.^13-15^ While the average connection comprises only 3-4 synapses, some specific connections consist of over 2,000 individual synapses (Fig. 1a). This heavy tail in the distribution of connection strength is highly non-random. Shuffling the placement of synapses destroys the strong connections, even after accounting for the variability between neurons (Fig. 1a). Heavy-tailed connectivity is not limited to a single neuron type or brain region, but instead arises consistently across the entire brain (Fig. 1b). Similar distributions appear in the combined brain and nerve cord of the female fly (Fig. 1c),^9^ the nerve cord of the male fly (Fig. 1d),^10^ the nematode *C. elegans* (Fig. 1e),^3, 4^ and the mouse retina (Fig. 1f),^5^ among other connectomes.^6, 11^ Even in measurements of activity (rather than structure), some rare pairs of neurons are correlated much more strongly than most.^12, 16-19^ Given the universality of heavy-tailed connections, does there exist an equally general purpose for neural function?

## Noisy connections induce errors in communication

Unlike artificial networks, in which weights are fixed with arbitrary precision, communication between neurons must contend with random fluctuations across many scales. Two neurons performing the same function in genetically identical organisms, or within the same organism, form different connections.^20-22^ Over time, individual neurons create new synapses, prune old synapses, and strengthen connections via plasticity.^23-25^ Even with connectivity held fixed, the response of a neuron can change due to stochastic ion channels,^26^ variable neurotransmitter release,^27, 28^ and thermal noise.^2, 29^ Thus, in the language of information theory, synaptic connections function as noisy communication channels between upstream inputs and a downstream output.^30^ Yet in the face of significant synaptic noise, key neural functions remain intact.^2, 31-33^

To investigate how synaptic noise impacts communication, consider a minimal McCulloch-Pitts (MP) neuron or perceptron (Fig. 2a),^34, 35^ which receives inputs from n presynaptic neurons (i=1,…,n) that are either active ($x_{i}=1$) or silent ($x_{i}=-1$). These inputs are weighted by connection strengths $w_{i}$, combined with a bias b, and then passed through a threshold to produce the output $y=\text{sgn}\;(\sum_{i}w_{i}x_{i}+b)$. Although a simplification of real neurons, this model captures key features of neural interactions and forms the basis for artificial networks.^19, 36-38^ Biological noise leads to random fluctuations ${\hat{w}}_{i}$ in the weights, such that the instantaneous strength of a connection is given by ${\tilde{w}}_{i}=w_{i}+{\hat{w}}_{i}$ (Fig. 2a). For example, if each unit of connection strength (e.g., each synapse or unit of contact area) is subject to independent noise of strength σ, then connections fluctuate according to $\text{Var}({\hat{w}}_{i})=\sigma^{2}w_{i}$. In turn, these fluctuations produce errors in communication: for the same inputs $x=\{x_{i}\}$, the postsynaptic neuron may generate a different output $\tilde{y}=\text{sgn}\;(\sum_{i}{\tilde{w}}_{i}x_{i}+b)$. Geometrically, the original neuron with weights $w=\{w_{i}\}$ defines a decision boundary in the space of inputs (Fig. 2b).^39^ Noise in the weights shifts this boundary, and any inputs x that cross the hyperplane result in errors $y\neq \tilde{y}$. Across all inputs and fluctuations, the neuron admits errors at a rate $ℰ=E[1(y\neq \tilde{y})]$, where 1(·) is the indicator function and E[⋅] represents an average over both inputs and noise. Importantly, the error rate depends only on the nature of the communication channel itself, which is determined by the strengths of connections.

To understand how connections shape errors, an important quantity is the local field $z=\sum_{i}w_{i}x_{i}+b$, which dictates whether the neuron fires (z>0) or not (z<0).^40^ To produce an error, the perturbation to the local field $\hat{z}=\sum_{i}{\hat{w}}_{i}x_{i}$ must be large enough to change the sign of z (Fig. 2c). In the simplest scenario, the bias b is zero and the inputs are independent and symmetric $\left(E(x_{i})=0\right)$. As the number of inputs n grows, the distributions of local fields z and perturbations $\hat{z}$ are defined exactly by the mean 〈w〉 and variance Var(w) of the weights (Fig. 2c; Methods). Increasing the variance pushes the local field away from the decision boundary (z=0), such that errors require stronger perturbations. Thus, for fixed average strength 〈w〉, as connections become more heterogeneous, the neuron admits fewer errors (Fig. 2d). In the limit n→∞, we arrive at an analytic prediction for the error rate,

(1) $$ℰ(w)=\frac{1}{\pi }\;\arctan\left(\sqrt{\frac{\sigma^{2}\langle w\rangle }{\text{Var}(w)+{\langle w\rangle }^{2}}}\right),$$

which we confirm in simulations (Fig. 2e). The error rate depends non-monotonically on the average strength 〈w〉: for low variance Var(w), errors are minimized by strong connections, while for high variance, weak connections minimize errors. By contrast, for any average strength 〈w〉, increasing the variance leads to a monotonic decrease in errors (Fig. 2e).

Strikingly, Eq. (1) reveals that errors do not depend independently on the mean and variance in the weights, but instead through a single statistic $r(w)=\sqrt{\frac{\text{Var}(w)}{\langle w\rangle }+\langle w\rangle }$. Since ℰ(w)=1πarctan(∕rσ(w)), the statistic r(w) defines the strength of noise σ that a neuron can withstand before errors become significant; that is, it defines the neuron’s *robustness*. When r(w)≫σ, errors vanish (ℰ→0), and when r(w)≪σ, errors dominate (ℰ→1∕2). Plotting the error rate as a function of robustness, we find that all of the data in Fig. 2e collapse onto a single analytic curve (Fig. 2f). Even without many of the simplifying assumptions, the same structure remains. As long as the average output E(y) is held fixed, the error rate still decreases monotonically with robustness for arbitrary bias b and input averages $E(x_{i})$ (Fig. 2g; Methods). One can also include correlations between inputs, continuous inputs and outputs, and different noise structures $\text{Var}({\hat{w}}_{i})$ (Supplementary Information). In all cases, increasing the variance in connection strengths pushes the neuron away from the decision boundary, thereby reducing errors in communication.

## Heavy-tailed connectivity supports robust communication

Ultimately, we seek to understand how real distributions of connection strength impact communication (Fig. 1). Across the fruit fly brain, neurons are highly heterogeneous, varying by over three orders of magnitude in the number of inputs n (Fig. 3a), one order of magnitude in average connection strength 〈w〉 (Fig. 3b), and four orders of magnitude in variance Var(w) (Fig. 3c).^7, 8^ For each neuron, we construct a synthetic MP neuron with weights $w_{i}$ defined by the number of synapses in each incoming connection (Fig. 2a), which is a reasonable approximation in the case of the fruit fly.^13-15^ We then simulate activity before and after adding noise to every synapse. Due to the heterogeneity between neurons, the same amount of synaptic noise induces a wide range of errors; yet despite this variability, errors are consistently predicted by the analytic rate in Eq. (1) (Fig. 3d). Grouping neurons based on their average connection strength 〈w〉, we find that errors systematically decrease with the variance in weights (Fig. 3e). Moreover, these errors are completely characterized by the robustness of each neuron r(w), such that over 10^5^ neurons across the entire brain collapse to one curve (Fig. 3f). This collapse—and our analytic predictions—hold consistently for different firing rates (Fig. 3g), amounts of synaptic noise (Fig. 3h), and definitions of the weights $w_{i}$ (Supplementary Information).

Across different organisms and brain regions, the fact that connections are heavy-tailed suggests that neuronal communication may be highly robust to synaptic noise (Fig. 1).^11^ To test this hypothesis, a natural comparison is a null neuron with the same total number of inputs and synapses (such that the average connection strength 〈w〉 is fixed), but with synapses shuffled among connections. These null neurons have Poisson distributions of connection strength, characterized by Var(w)=〈w〉. In the fly brain, we see that most neurons have variance larger than Poisson, some by multiple orders of magnitude (Fig. 4a, *inset*).^7, 8^ These highly variable weights, punctuated by abnormally strong connections, make the vast majority of neurons (93%) more robust than their shuffled counterparts (Fig. 4a).

We repeat the same analysis for many neurons spanning different individuals, systems, and species. In the combined brain and nerve cord of the female fruit fly, a staggering 97% of neurons are more robust to noise than comparable nulls (Fig. 4b).^9^ This percentage drops slightly in the nerve cord of the male fly (95%)^10^ and more dramatically in *C. elegans* (74%; Fig. 4b),^3, 4^ reflecting limitations on very strong connections in the nematode (Fig. 1e).^11^ In vertebrates, connections are strengthened by increasing not only the number of synapses, but also the sizes of individual synapses.^41, 42^ Thus, for neurons in the mouse retina, we construct synthetic neurons with weights $w_{i}$ defined by the total contact area with each partner (Fig. 1f).^5^ After shuffling contact area among connections, thereby destroying the heavy-tailed connectivity, we find that 99.5% of neurons become less robust to noise (Fig. 4b). Even within a single organism, across all regions of the fly brain, the majority of neurons are highly robust (Fig. 4d).^7, 8, 43^ Together, these results suggest that heavy-tailed connectivity, which is observed universally in biological networks, supports reliable communication in the face of synaptic noise.

## Robustness across an adult brain

In the fruit fly, we have seen that robustness varies dramatically from one neuron to another (Fig. 4a-b).^7-10^ Is this robustness distributed evenly throughout the brain, or instead, can some regions or cell types withstand more noise than others? To compare across cells, we need a standardized scale that accounts for differences in average connection strength 〈w〉. For any neuron, robustness is minimized by spreading synapses uniformly among inputs, yielding $r_{\text{min}}(w)-\sqrt{\langle w\rangle }$. By distributing synapses randomly, robustness increases to $r_{\text{rand}}(w)=\sqrt{1+\langle w\rangle }$. This difference defines a natural scale, leading to a normalized robustness,

(2) $$\hat{r}(w)=\frac{r(w)-r_{\text{min}}(w)}{r_{\text{rand}}(w)-r_{\text{min}}(w)}.$$

Any normalized robustness above (below) one means that synapses are distributed to withstand more (less) noise than random. Indeed, after shuffling synapses among connections, we see that normalized robustness peaks sharply at $\hat{r}(w)=1$ (Fig. 5a). By contrast, real neurons exhibit a much wider range of robustness, with the median neuron 3.4 times more robust than random.

The fly brain naturally partitions into distinct anatomical regions, which are responsible for different neural functions.^7, 8, 43^ Because neurons can have incoming or outgoing synapses in different brain regions, we assign each neuron to the region that contains the most of its incoming synapses (Methods). Between regions, we find that robustness varies significantly, increasing by an order of magnitude from the least robust region (ocelli) to the most robust (lateral complex; Fig. 5b). Notably, a hierarchy emerges in which regions involved in sensory processing (e.g., antennal and optic lobes)^49, 50^ are less robust than those associated with central functions (e.g., mushroom body and central complex).^51, 52^

To understand whether robustness increases from the sensory periphery to the central brain, we define the distance of each neuron from an initial set of sensory cells using a spreading process (Methods).^7, 8, 44^ For example, vision begins at the photoreceptors R1-8,^53^ which, due to their small number of weak synaptic inputs, have normalized robustness near zero (Fig. 5c, d). As we move away from the periphery, robustness increases dramatically to over seven times higher than random, before returning to the whole-brain median (Fig. 5c). We observe a similar pattern in specific visual circuits, such as the ON motion pathway, which detects increases in light intensity.^45, 46^ Robustness rises abruptly from the photoreceptors R1-6 to downstream interneurons L1 and Mi1, and decreases to the median at the outputs T4 (Fig. 5d; *grey*). The OFF pathway, which detects decreases in light intensity, displays a nearly identical pattern (Fig. 5d; *orange*). Comparatively, olfaction begins at receptor neurons (ORNs), which have normalized robustness near one, equivalent to shuffled synapses (Fig. 5e, f). As signals propagate from the periphery, neurons again become multiple times more robust before settling to the median (Fig. 5e). Along the odor encoding pathway,^47^ robustness increases sharply from ORNs to projection neurons (PNs), Kenyon cells (KCs), and the eventual outputs in the mushroom body (MBONs; Fig. 5f). Thus, in both vision and olfaction, neurons become more robust with distance from the sensory periphery.

From the periphery to the central brain, circuits transition from feedforward to highly recurrent, suggesting that robustness may be linked to network structure.^48, 54^ Locally, recurrence is quantified by a neuron’s reciprocity: the fraction of neighbors that are both pre- and postsynaptic partners.^48^ Across the fly brain, as neurons become less feedforward and more reciprocal, they consistently become more robust (Fig. 5g), reinforcing the association between central processing and robustness to synaptic noise. Finally, in addition to anatomical structure and network topology, neuronal interactions are driven by the identity of the neurotransmitter released by each neuron.^7, 8, 55^ We find that neurons involved in both fast excitation and inhibition—releasing GABA, acetylcholine (ACH), and glutamate (GLUT)—all exhibit similar distributions of robustness (Fig. 5h).^55^ By contrast, modulatory neurons that release serotonin (SER) and dopamine (DA) are significantly less robust, while those that release octopamine (OCT)—which mediates memory, arousal, and behavioral state—are more robust.^56-58^ Thus, the significant variation in robustness between neurons is not random, but instead couples tightly to neural function and structure.

## Discussion

Connections between neurons are universally heavy-tailed (Fig. 1),^3-11^ hinting at a fundamental role for neural function. Here we show that in the presence of noise, heavy-tailed connections reduce errors in communication. In synthetic neurons, a single network statistic (robustness) sets the scale at which noise leads to errors (Fig. 2). Even for the same average connection strength, as distributions grow wider, neurons become more robust to noise, thereby suffering fewer errors. Leveraging advances in high-throughput connectomics, we investigate the robustness of hundreds of thousands of neurons spanning several species and neural systems (Figs. 3-5). By concentrating synapses among strong connections, most neurons are highly robust, suggesting that connectivity may be optimized to overcome biological noise.

Our results and their limitations point to concrete future directions. To make analytic progress [Eq. (1)], we employ synthetic models of MP neurons or perceptrons (Fig. 2a). While these models capture key features of neural interactions and have been used to predict neural activity,^19, 36-38, 59^ real neurons involve complex biophysical processes that induce interactions between inputs.^60, 61^ Building upon our framework, one can study robustness in more realistic models of neural activity. This will shed light on how robust communication is shaped not only by synapse count and strength, but also by synapse location, neuron morphology, and other biophysical parameters. Importantly, as long as noise has a sub-multiplicative impact on a neuron’s local field, we still expect heavy-tailed connectivity to reduce errors in communication (Supplementary Information).

Although we focus on individual neurons as stochastic communication channels, signals in the brain propagate through complex networks that involve many neurons.^54^ By wiring neurons together and simulating collective dynamics, one can investigate how synaptic noise leads to errors at the network level.^2^ In turn, this will reveal how different circuit structures support robust communication and optimize information flow.^2, 54^ Finally, our results indicate that distinct parts of the brain are structured to withstand different amounts of noise (Fig. 5), yet the reasons remain unclear. Sensory neurons may be optimized for sensitivity to small changes in inputs at the expense of robustness.^62^ At the same time, signaling in the central brain may be forced to contend with greater noise than at the periphery.^2^ As experiments continue to advance, identifying universal features of network structure and understanding how they support neural functions remain critical outstanding challenges.

## Methods

### Connectome data.

The datasets used in this paper were previously published and are openly accessible.^3-5, 7-10^ All connectomes were sectioned and imaged using electron microscopy. Identification and classification of neurons and synapses were achieved via a combination of manual labeling and automated processing with subsequent proofreading. We provide the preprocessing code required to reproduce the results from the original datasets (Data Availability). Connections in the mouse retina correspond to contact area between neurons, and are undirected.^5^ For our analysis, we treat each contact as an incoming connection to both neurons involved. The connectome for the female adult fly brain (FAFB) identifies each synapse with pre- and postsynaptic neurons as well as the neuropil in which it is located, which are grouped into brain regions.^7, 8, 43^ We identify connections with brain regions based on which region contains the plurality of their synapses (for Fig. 1b) and analogously assign neurons based on plurality of their post-synapses (for Figs. 4d and 5b). Roughly 95% of connections consist of synapses within a single brain region, and 70% of neurons have all incoming synapses in a single brain region (Supplementary Information). We assign neurotransmitters to neurons following the FAFB classification.^7, 8^

### Simulated error rate.

Unless otherwise stated, simulations use zero bias (b=0), inputs $x_{i}$ are drawn independently and uniformly from {−1, +1}, weight perturbations ${\hat{w}}_{i}$ are drawn from a zero-mean Gaussian with noise strength parameter σ=1, and the error rate for each neuron is averaged over all combinations of 10^3^ input samples and 10^3^ weight perturbations. All synthetic neurons have $n=10^{3}$ inputs. In Fig. 2d, we generate three neurons: one with uniform weights $\left(w_{i}=1\right)$ and two with gamma-distributed weights of mean 〈w〉=1 and variances Var(w)=0.8 and 3. The histograms use the data from all combinations of 5 × 10^3^ input samples and 5 × 10^3^ weight perturbations. Each data point in Figs. 2e-g represents an average over 10^3^ neurons with gamma-distributed weights with a specified mean and variance. In Fig. 3h, we adjust the noise strength σ. In Figs. 2g and 3g, we change the firing rate by tuning the bias b using the Gaussian approximation in Eq. (3). In the Supplementary Information, we investigate the effects of sparse inputs $\left[E(x_{i})<0\right]$, correlations between inputs, different noise structures, and different definitions of connection strength.

### Analytic error rate.

To derive the analytic prediction for the error rate, we study the limit of many inputs n→∞. For a set of weights $w=\{w_{i}\}$ and bias b, the local field is $z=\sum_{i}w_{i}x_{i}+b$. Given noise $\hat{w}=\{{\hat{w}}_{i}\}$, the perturbed weights $\tilde{w}=w+\hat{w}$ yield a perturbed local field $\tilde{z}=\sum_{i}{\tilde{w}}_{i}x_{i}+b$. Errors occur when the signs of z and $\tilde{z}$ differ, such that the error rate is given by $ℰ=E[1(\text{sgn}(\tilde{z})\neq \text{sgn}(z))]$, where E(⋅) is an expectation over realizations of the inputs x and noise $\hat{w}$.

To study the n→∞ limit, we assume that weights $w_{i}$ are drawn independently from a distribution P(w) with finite mean and variance; here, 〈⋅〉 denotes an average over P(w). We assume the inputs $x_{i}$ are independent and identically distributed for each n; this distribution can change as n grows, but must converge. We also allow the bias b to change. As n increases, we hold the output firing probability fixed to a finite value $p_{f}$. For large n, the central limit theorem dictates that

(3) $$p_{f}=\Phi \left(\frac{\mu_{z}}{\sigma_{z}}\right),$$

where Φ(⋅) is the standard normal cumulative distribution function and $\mu_{z}$ and $\sigma_{z}^{2}$ are the mean and variance of z, respectively. Moreover, the correlation between z and $\tilde{z}$ is given by

(4) $$\rho ={\left(1+\frac{\sigma^{2}}{\sigma_{x}^{2}}\frac{\langle w\rangle }{\langle w^{2}\rangle }\right)}^{-1∕2}={\left(1+{\left(\frac{\sigma }{\sigma_{x}r(w)}\right)}^{2}\right)}^{-1∕2},$$

where $r(w)=\sqrt{\frac{\text{Var}(w)}{\langle w\rangle }+\langle w\rangle }$ is the robustness of the neuron and $\sigma_{x}^{2}$ is the input variance.

The distribution of normalized local fields $\left(z∕\sigma_{z},\tilde{z}∕\sigma_{\tilde{z}}\right)$ converges to a Gaussian with means $E(z∕\sigma_{z})=\Phi^{-1}(p_{f})$ and $E(\tilde{z}∕\sigma_{\tilde{z}})=\rho \Phi^{-1}(p_{f})$, unit variance, and covariance ρ. Computing the error rate amounts to computing the mass in the upper-left and lower-right orthants. The solution to the Gaussian orthant problem yields

(5) $$ℰ=p_{f}+\Phi (\rho \Phi^{-1}(p_{f}))-2\Phi_{2}(\Phi^{-1}(\rho_{f}),\rho \Phi^{-1}(\rho_{f});\rho ),$$

where $\Phi_{2}$ is the bivariate standard normal cumulative distribution function (Supplementary Information). We have therefore established that the error rate depends only on the output firing probability $p_{f}$ and the correlation between local fields ρ, which itself depends on the robustness r(w), noise σ, and input variability $\sigma_{x}$. Thus, the weights $w_{i}$ only enter the error rate through the firing probability and robustness. For a firing rate $p_{f}=1∕2$, the error rate simplifies significantly to Eq. (1).^64^

### Grouping neurons by connectivity statistics.

In Figs. 3e-h, we group neurons based on the statistics of their incoming connections. For each neuron, we compute the average number of synapses and the variance in the number of synapses among incoming connections. Since both average strength and variance in strength are approximately log-normally distributed (Figs. 3b-c), we first group neurons into uniformly log-spaced bins by average strength. Then within each bin, we group neurons into deciles by variance in strength. Each point in Figs. 3e-h represents the median over neurons in one group.

### Shuffled neurons.

We generate one shuffled neuron for each real neuron with at least ten incoming connections (Fig. 4). For the female fruit fly brain,^7, 8^ fruit fly male nerve cord,^10^ and nematode *C. elegans*,^3, 4^ we randomly shuffle synapses among incoming connections. The fruit fly brain and nerve cord connectome only includes connections with at least three synapses.^9^ To generate shuffled neurons, we first assign three synapses to each incoming connection and then place the remaining synapses randomly. For the mouse retina,^5^ connection strength is functionalized as the total contact area across all contacts comprising one connection, such that the measure of connection strength is no longer discrete. To generate shuffled neurons, we sample uniformly from the set of connection strength vectors w with total contact area $\sum_{i}w_{i}$ matching the original neuron. See the Supplementary Information for alternative shuffling choices.

### Distance to periphery.

For each neuron in the female fruit fly brain, to define the distance from a set of sensory neurons (Fig. 5c, e), we use a network spreading process introduced in Ref. 44. The process begins by defining a seed set of neurons. At each time step, downstream neurons are probabilistically added to the set. Specifically, each neuron i in the set succeeds in adding each outside neuron j with probability $p_{ij}=\text{min}\left\{\frac{1}{q}\frac{w_{ij}}{\sum_{k}w_{kj}},1\right\}$, where $w_{ij}$ is the number of synapses from i to j and q=0.3 is a threshold.^44^ For each seed set, we simulate the spreading process 10^3^ times. Simulations are terminated when all neurons in the brain are reached, or if no neurons are added in ten consecutive steps. Starting from the visual periphery, the process reaches an average of 98.9% of neurons, while an average of 98.6% are reached when starting from the olfactory periphery. We define the distance from neuron i to the periphery as the average number of simulation steps required to add i to the set. We then group neurons into quantiles of distance to the periphery.

### Reciprocity.

For a given neuron i, we define $N_{\text{up}}^{i}$ and $N_{\text{down}}^{i}$ to be the set of neurons immediately upstream or downstream of i, respectively. The reciprocity of i is the fraction of all neighbors that are both upstream and downstream, $|N_{\text{up}}^{i}\cap N_{\text{down}}^{i}|∕|N_{\text{up}}^{i}\cup N_{\text{down}}^{i}|$ (Fig. 5g). Reciprocity is bounded between 0 (fully feedforward) and 1 (fully recurrent).

## Supplementary Material

1

**Supplementary Information.** Supplementary text and figures accompany this paper.

## Figures and Tables

**Fig. 1 ∣:**
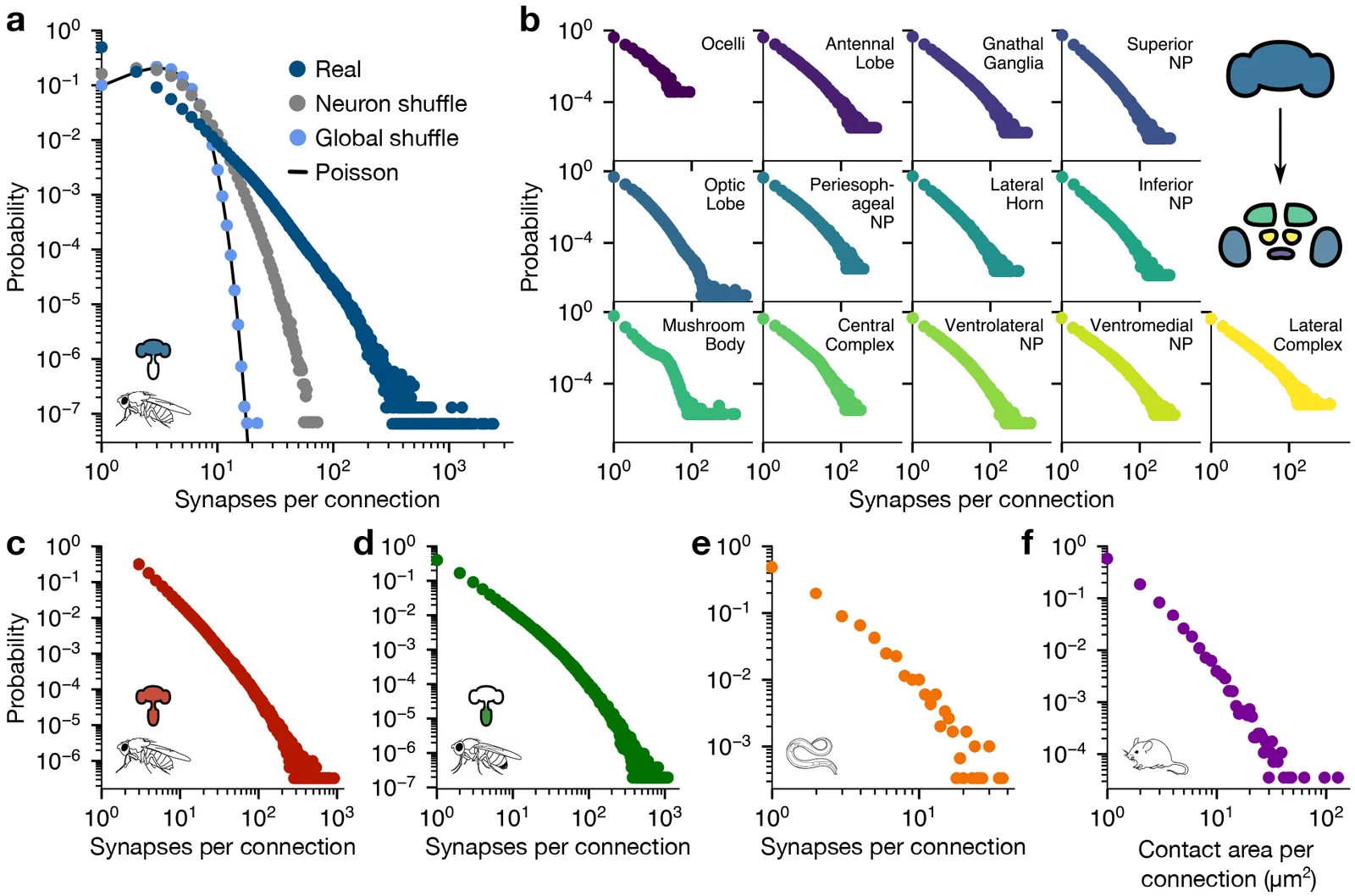
Heavy-tailed synaptic connectivity across species and systems. **a**, Distribution of synapses per connection between neurons in the whole brain of the female fruit fly *Drosophila*^7, 8^ (dark blue), after shuffling the synapses among incoming connections to each neuron (grey) and after shuffling synapses among all connections in the network (light blue); solid line indicates a Poisson distribution. **b**, Distributions of synapses per connection for different regions of the female fruit fly brain.^7, 8^ Connections are assigned to regions based on the plurality of synapses (Methods). **c-e**, Distributions of the number of synapses per connection in the combined brain and nerve cord of a *Drosophila* female (**c**),^9^ the *Drosophila* male nerve cord (**d**),^10^ and the nematode *C. elegans* (**e**).^3, 4^ **f**, Distribution of the total contact area (rounded to the nearest *μ*m^2^) per connection between neurons in the inner plexiform layer of the mouse retina.^5^

**Fig. 2 ∣:**
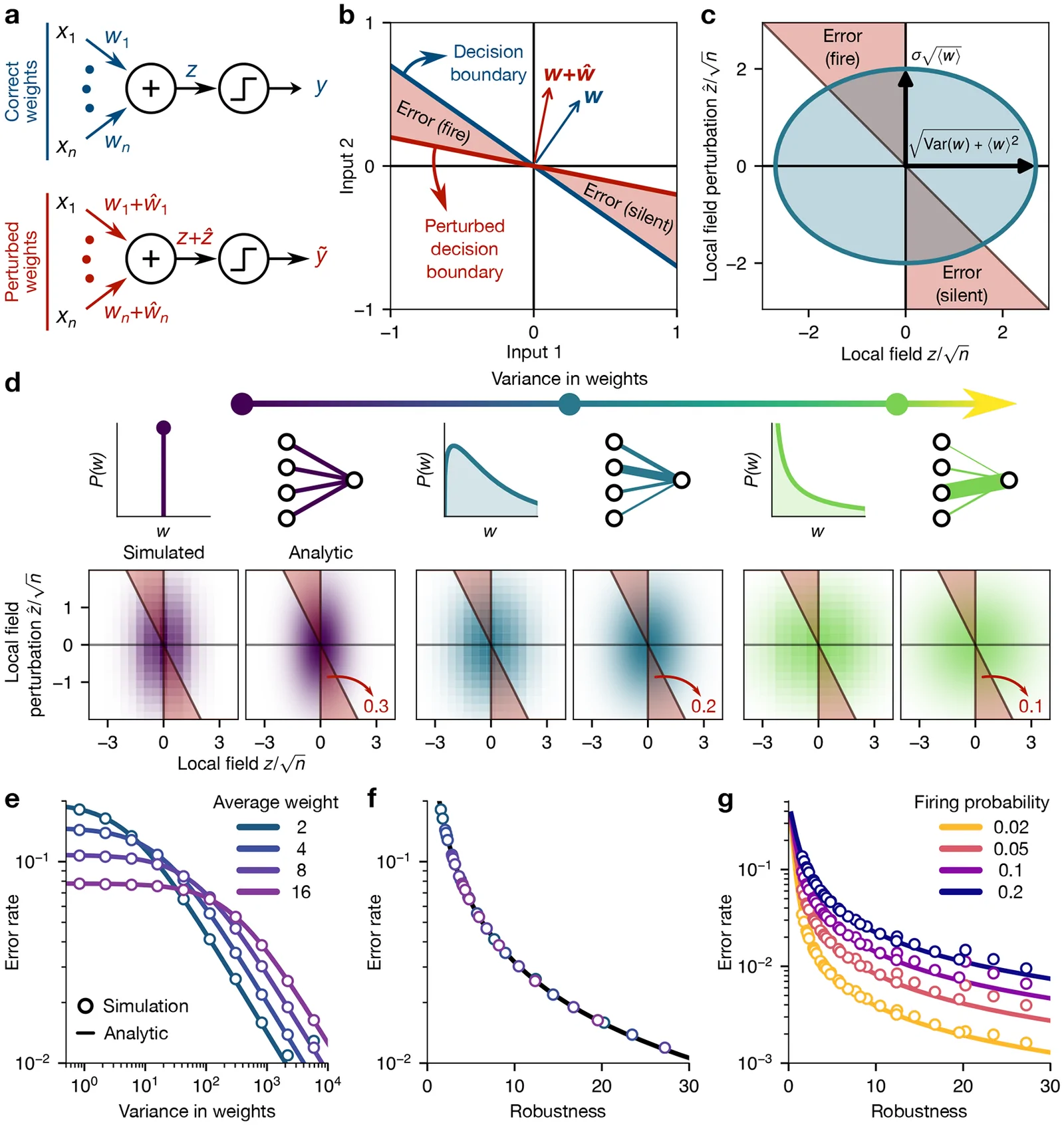
Quantifying errors due to synaptic noise. **a**, Minimal model of noise in neural communication. *Top:* In a McCulloch-Pitts (MP) neuron or perceptron, the neuron passes n inputs $x_{i}$ through linear weights $w_{i}$ to form a local field z, which determines the binary output y=sgn(z). *Bottom:* Noise ${\hat{w}}_{i}$ in the weights yields a perturbed local field $z+\hat{z}$, which can lead to a different output $\tilde{y}\;=\;\text{sgn}(z+\hat{z})$. **b**, Synthetic neurons define decision planes in the high-dimensional input space. Noise in the weights shifts the boundary, and inputs that cross the hyperplane represent errors $\tilde{y}\neq y$. **c**, Joint distribution of the local field z and perturbation $\hat{z}$ determines the error rate. For independent inputs, as n→∞ the distribution approaches a Gaussian with widths defined by the mean and variance of the weights (Methods). Blue region illustrates a level set of the distribution with b=0 and $E(x_{i})=0$, and arrows represent standard deviations. **d**, Simulated and analytic distributions of local fields z and perturbations $\hat{z}$ for three neurons with $n=10^{3}$ inputs and noise of strength σ=1. From left to right, incoming weights are drawn from distributions with equal mean and increasing variance, leading to decreasing errors (Methods). Color is proportional to the probability density. Inset numbers indicate error rates. **e**, Error rates for synthetic neurons with fixed average connection strength and increasing variance. **f**, Error rate as a function of robustness r(w) for the same neurons as panel **e**. **g**, Error rate as a function of robustness for neurons with the same weights as panels **e** and **f**, but different firing probabilities (Methods). In **e-g**, each neuron has $n=10^{3}$ inputs, noise of strength σ=1, and weights drawn from gamma distributions with fixed mean and variance. Lines are analytic predictions and points are averages over simulations of 10^3^ neurons (Methods).

**Fig. 3 ∣:**
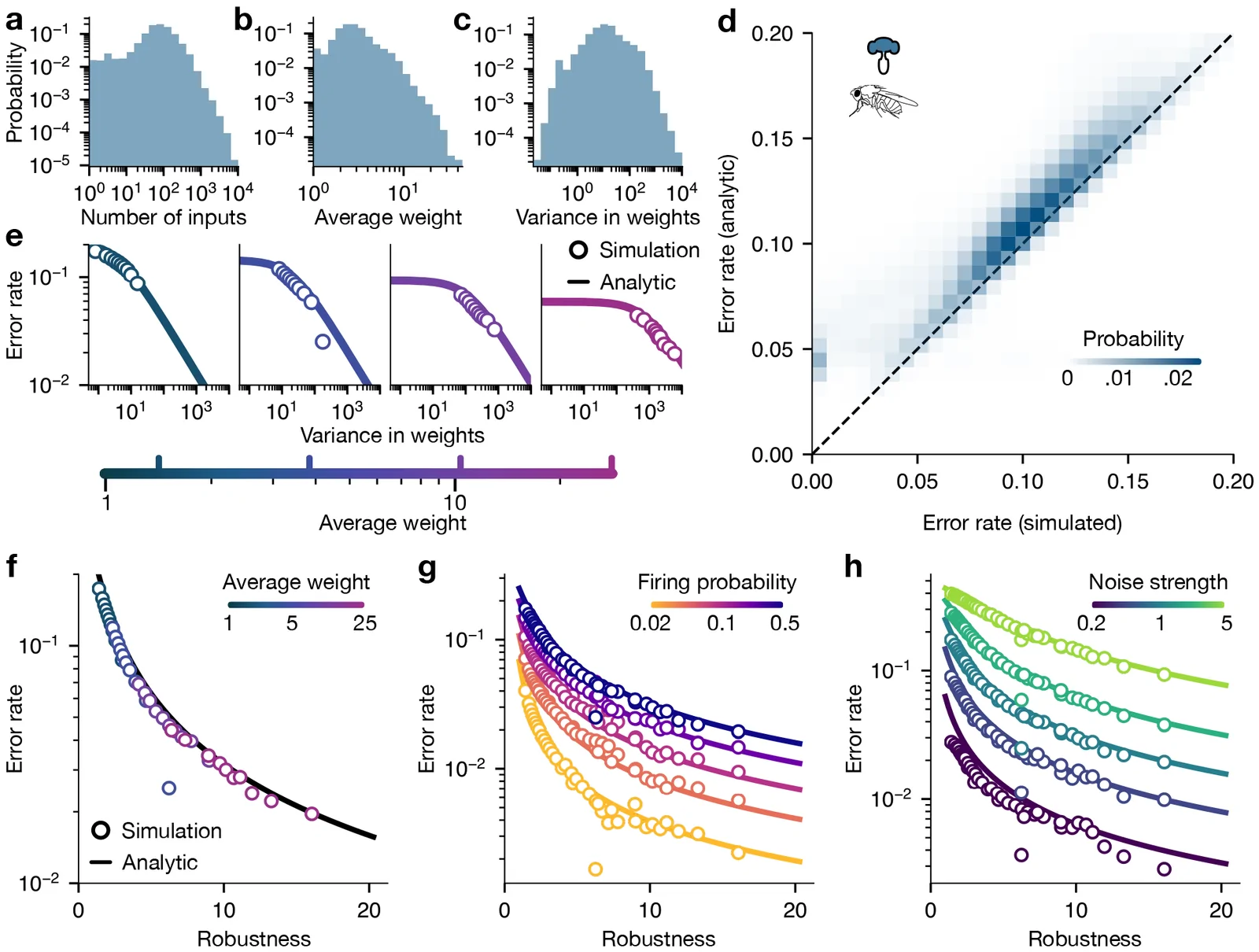
Communication errors with real connection strengths. **a-c**, Distributions of numbers of inputs n (**a**), average connection strength 〈w〉 (**b**), and variance in connection strengths Var(w) (**c**) across neurons in the whole brain of the female fruit fly.^7, 8^ **d**, For each neuron in the fly brain, we construct a synthetic neuron with weights defined by the number of synapses with presynaptic partners. Distribution of simulated and analytic error rates across all neurons with at least ten inputs. Unless otherwise specified, bias is zero (b=0), inputs are independent and symmetric ($E(x_{i})=0$), and synaptic noise is applied with strength σ=1. **e**, Error rates as a function of variance in connection strengths for increasing average strength. Neurons are grouped by the mean and variance of their incoming connection strengths (Methods). Points represent the median values within each group, and solid lines are analytic predictions from Eq. (1). **f**, Error rate as a function of robustness r(w) for the groups of neurons in **e**. **g-h**, Error rate as a function of robustness for the same groups with different firing rates (**g**) and different levels of synaptic noise (**h**). See Methods for simulation details.

**Fig. 4 ∣:**
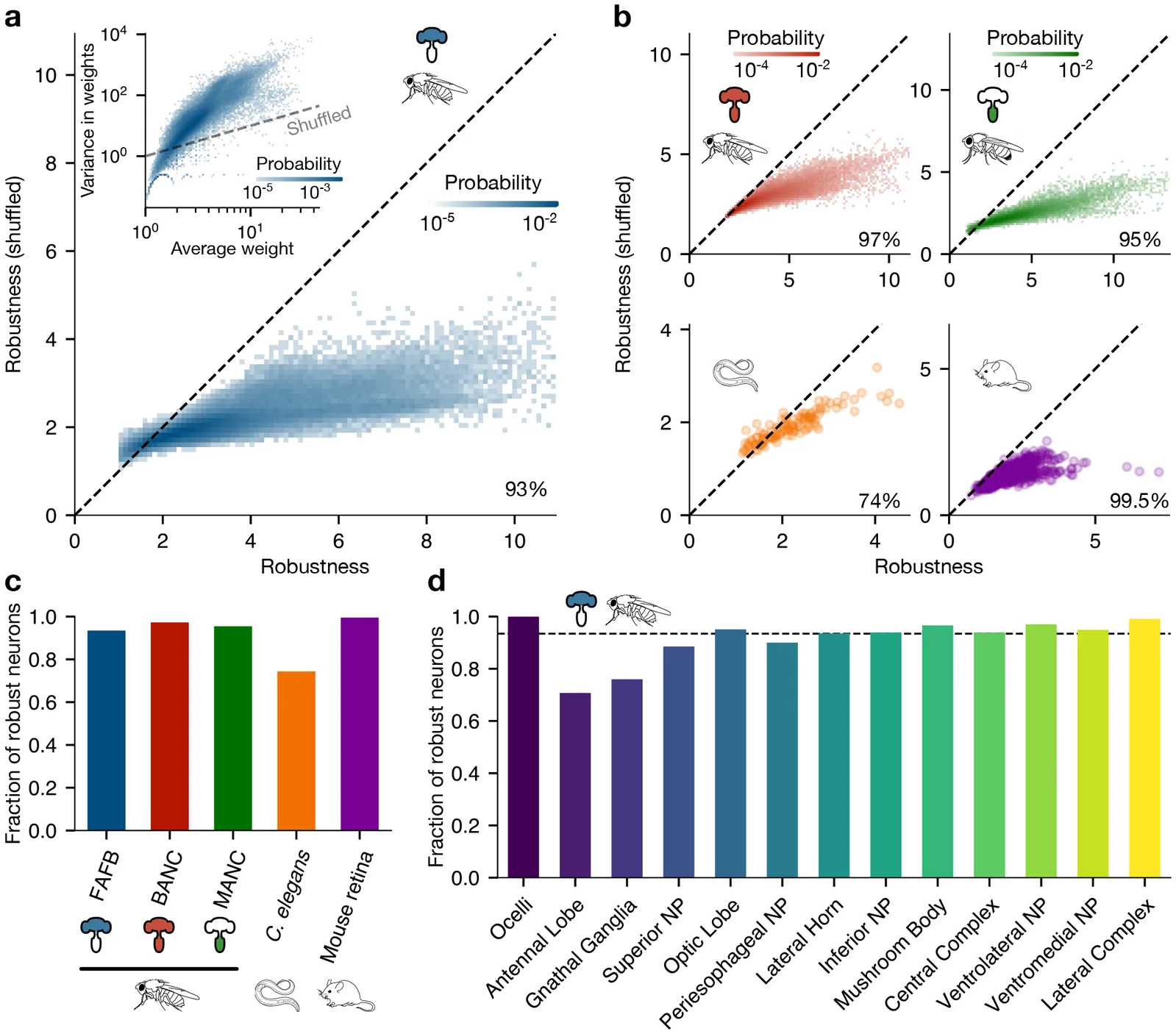
Robust communication driven by heavy-tailed connectivity. **a**, Distribution of robustness r(w) across neurons in the female fruit fly brain^7, 8^ before and after shuffling synapses among incoming connections. Percentage indicates fraction of neurons below the dashed line, which are more robust than shuffled nulls. *Inset:* Distribution of average connection strength 〈w〉 and variance in strengths Var(w) over neurons in the female fly brain,^7, 8^ where each weight represents the number of synapses in an incoming connection. Dashed line represents the Poisson variance for shuffled neurons. **b**, Distribution of robustness before and after shuffling connection strength among incoming connections for neurons in the combined brain and nerve cord of a *Drosophila* female (*top left*),^9^ the nerve cord of a *Drosophila* male (*top right*),^10^ the nematode *C. elegans* (*bottom left*),^3, 4^ and the inner plexiform layer of the mouse retina (*bottom right*).^5^ Dashed lines represent equality, and percentages indicate the fraction of neurons with higher robustness than shuffled nulls (Methods). **c**, Fractions of neurons with robustness larger than shuffled counterparts in different connectomes. **d**, Fractions of neurons with robustness larger than shuffled nulls in different regions of the female fruit fly brain.^7, 8^ Dashed line represents the percentage for the whole brain. Neurons are assigned to brain regions based on the plurality of incoming synapses (Methods). In all connectomes, we only study neurons with at least ten incoming connections.

**Fig. 5 ∣:**
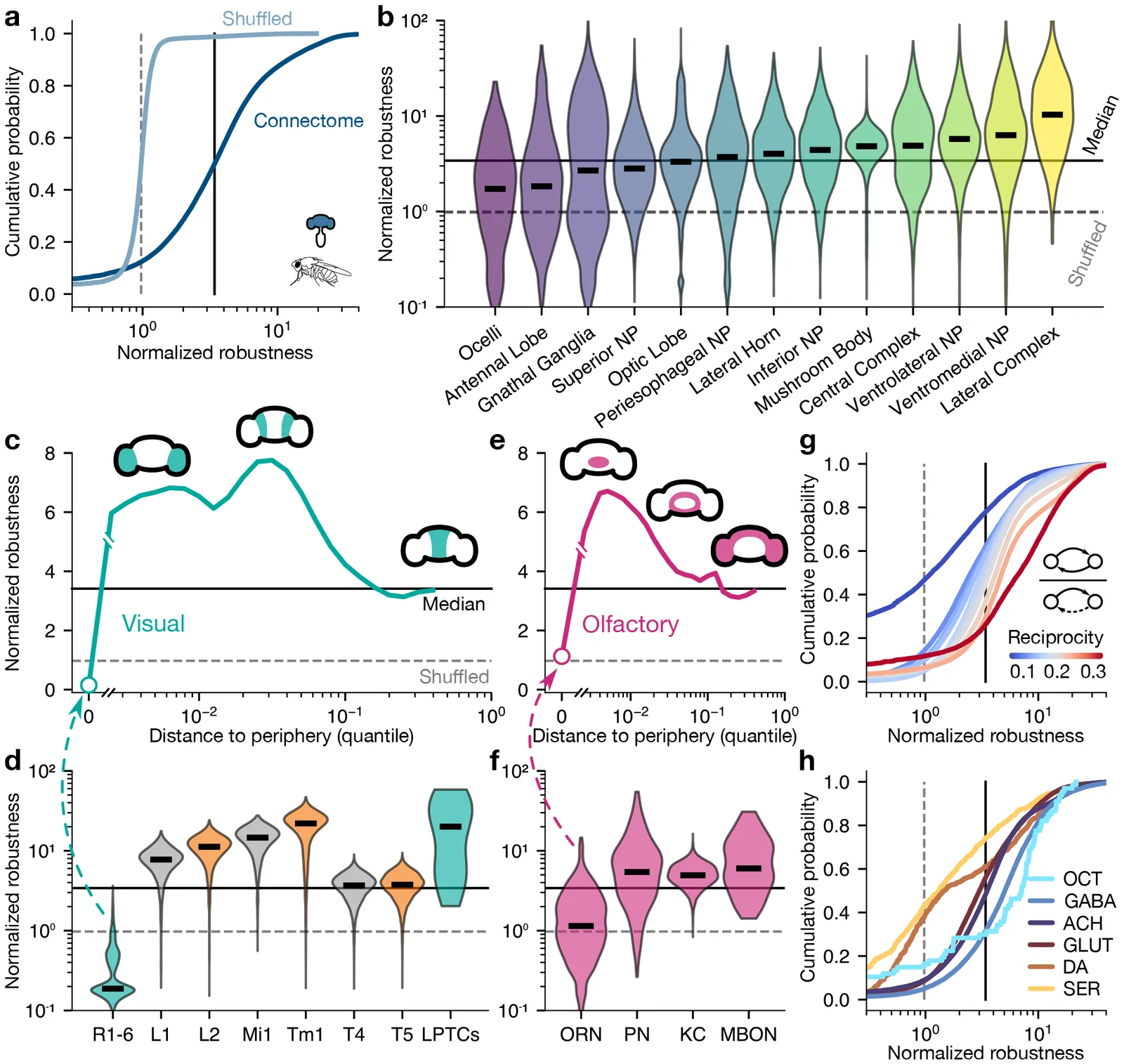
Robustness in the *Drosophila* brain. **a**, Cumulative distributions of normalized robustness across neurons in the fruit fly brain before and after shuffling synapses among incoming connections.^7, 8^ Lines indicate medians. **b**, Distribution of normalized robustness across neurons within each brain region. Neurons are assigned to brain regions based on the plurality of their incoming synapses (Methods). **c**, Normalized robustness versus distance from the visual periphery, calculated using a spreading process initialized at the photoreceptors R1-6 (Methods).^7, 8, 44^ Values represent medians among neurons grouped by their (log) quantile distance to the periphery. **d**, Distribution of normalized robustness for specific cell types along the ON (teal and grey) and OFF (teal and orange) motion pathways in the visual system.^45, 46^ LPTCs include HS, VS, H1/H2, and DCH/VCH neurons. **e**, Normalized robustness versus quantile distance from the olfactory periphery, calculated using a spreading process initialized at the olfactory receptor neurons (ORNs). **f**, Distribution of normalized robustness for specific cell types along the odor encoding pathway.^47^ **g**, Distributions of normalized robustness for neurons grouped by reciprocity, or the fraction of neighbors that are both pre- and postsynaptic partners (Methods).^48^ **h**, Distributions of normalized robustness for neurons grouped by neurotransmitter release (Methods). In all panels, dark solid lines represent the median normalized robustness across the entire fruit fly brain, and light dashed lines represent neurons with shuffled synapses.

**Table 1:** Statistics of neuronal connectomes

Statistic	*Drosophila* FAFB^7, 8^	*Drosophila* BANC^63^	*Drosophila* MANC^10^	*C. elegans* ^3, 4^	Mouse retina^5^
Number of neurons	138,639	153,962	23,641	279	840
Number of connections	1.51 × 10^7^	3.04 × 10^6^	5.31 × 10^6^	2.99 × 10^3^	1.30 × 10^5^
Incoming connections per neuron	108.9	19.7	224.4	10.7	154.2
Synapses per connection (contact area for mouse retina)	3.6	7.8	5.8	2.7	1.0 *μ*m^2^
Fraction of neurons with $k_{\text{in}}\geq 10$	0.900	0.456	0.981	0.419	0.990

## Data Availability

Raw connectome data analyzed in this study are previously published and are available as deposited at Zenodo (https://doi.org/10.5281/zenodo.23427196).

## Code Availability

The code used to perform the analyses in this paper is openly available at github.com/jbetancourt015/robust_communication.
