# Supplementary material for: Heavy-tailed connections support robust neural communication
Source: bioRxiv. 2026 Oct 26:2026.10.21.760834. Preprint. [Version 1] doi: 10.64898/2026.10.21.760834 (PMC13733683; doi:10.64898/2026.10.21.760834)
Supplement: Supplementary file 1 [file NIHPP2026.10.21.760834v1-supplement-1.pdf]

bioRxiv preprint doi: https://doi.org/10.64898/2026.10.21.760834; this version posted October 26, 2026. The copyright holder for this preprint (which was not certified by peer review) is the author/funder, who has granted bioRxiv a license to display the preprint in perpetuity. It is made available under a CC-BY 4.0 International license.

Supplementary Information

1. Introduction

In this Supplementary Information, we provide additional analyses, statistics and robustness checks to support the results in the main text. Throughout, we use $\mathbb{E}_u(\cdot)$ to denote the expectation value with respect to the random variable u; angle brackets $\langle\cdot\rangle$ instead denote empirical averages over a neuron's realized incoming connection weights. In Sec. 2, we provide an alternative derivation of the robustness result that makes use of the geometric intuition of classification planes in the space of inputs. In Sec. 3, we extend our results to sparse inputs and outputs, showing that the input weights still only enter the error rate through the robustness of the neuron. In Sec. 4, we give conditions under which robustness characterizes the error rate when inputs are correlated. In Sec. 5, we show that, under a more general class of noise structures, the error rate still depends on the input weights only via (a modified version of) robustness. In Sec. 6, we extend our analysis to continuous outputs, and show that the advantages of heavy-tailed connection strengths arise from the nonlinearity of neuronal responses. In Sec. 7, we demonstrate that our error rate prediction remains accurate under alternative definitions of connection strengths. In Sec. 8, we show that high robustness arises from the distributional structure of incoming connection strengths. In Sec. 9, we show how different choices of shuffling procedures affect robustness when connections are thresholded. In Sec. 10, we measure the fraction of synapses that get assigned the wrong brain region when connections and neurons are assigned to regions, and show that the majority of our coarse-grainings have a small fraction of misclassified synapses.

2. A geometric visualization of robustness

Our analysis of the error rate relied on analyzing the distribution of the local field z and its perturbations $\hat{z}$. We can build a complementary intuition by analyzing the effects of perturbations in input space.

Before we begin our analysis, we note that our derivation of the analytic prediction of the

bioRxiv preprint doi: https://doi.org/10.64898/2026.10.21.760834; this version posted October 26, 2026. The copyright holder for this preprint (which was not certified by peer review) is the author/funder, who has granted bioRxiv a license to display the preprint in perpetuity. It is made available under a CC-BY 4.0 International license.

error rate does not rely on the inputs being binary, only that they have zero mean. Therefore, our prediction for the error rate also holds for centered continuous inputs. This allows us to establish a clean benchmark for our geometric analysis: Gaussian inputs. Once we have built intuition for this benchmark, we can apply it to our binary input framework.

We consider inputs drawn from a multivariate Gaussian:

$$\boldsymbol{x} \sim \mathcal{N}(\boldsymbol{0}, \boldsymbol{I}_n), \tag{6}$$

where $\boldsymbol{I}_n$ is the identity matrix of dimension n. Given this distribution of inputs, we can ask: what is the probability of an error for weights $\boldsymbol{w}$ and a given set of perturbed weights $\tilde{\boldsymbol{w}}$? The answer is that the error is proportional to the angle θ between the perturbed and unperturbed weights (Eq. 4.12 in Ref. 65):

$$\mathbb{P}\{y \neq \tilde{y} | \tilde{\boldsymbol{w}}\} = \frac{\theta}{\pi}, \quad \theta = \arccos\left(\frac{\boldsymbol{w} \cdot \tilde{\boldsymbol{w}}}{\|\boldsymbol{w}\| \|\tilde{\boldsymbol{w}}\|}\right). \tag{7}$$

This is easy to visualize in the case of two inputs. Consider first only the inputs at a distance R. Given the rotational symmetry of the Gaussian, the distribution of inputs at a given distance is uniform. Therefore, the probability of an error given that the input is at a distance R is the fraction of the circle on which errors occur, which is $2\theta/2\pi = \theta/\pi$ (Fig. S1a). For n dimensions, we can compute this probability by segmenting space into planes parallel to the plane formed by $\boldsymbol{w}$ and $\tilde{\boldsymbol{w}}$. Conditional on the input lying on one of these planes, we can use an argument analogous to the two-dimensional one to show that the error probability is θ/π. Since this is true for any plane, it is true for all space, and we obtain the general result in Eq. (7).

We now have a geometric interpretation for what is driving the errors for a fixed perturbation. How do we extend this interpretation to the error rate? To obtain the error rate, we have to average over all possible $\tilde{\boldsymbol{w}}$:

$$\mathcal{E}(\boldsymbol{w}) = \mathbb{E}_{\tilde{\boldsymbol{w}}}\left(\mathbb{P}\{y \neq \tilde{y} | \tilde{\boldsymbol{w}}\}\right) = \frac{1}{\pi} \mathbb{E}_{\tilde{\boldsymbol{w}}}\left(\arccos\left(\frac{\boldsymbol{w} \cdot \tilde{\boldsymbol{w}}}{\|\boldsymbol{w}\| \|\tilde{\boldsymbol{w}}\|}\right)\right). \tag{8}$$

Since we are interested in the large-n behavior of the error rate, it is useful to re-write the expression above in terms of averages rather than sums, since we can expect these not to scale with n.

bioRxiv preprint doi: https://doi.org/10.64898/2026.10.21.760834; this version posted October 26, 2026. The copyright holder for this preprint (which was not certified by peer review) is the author/funder, who has granted bioRxiv a license to display the preprint in perpetuity. It is made available under a CC-BY 4.0 International license.

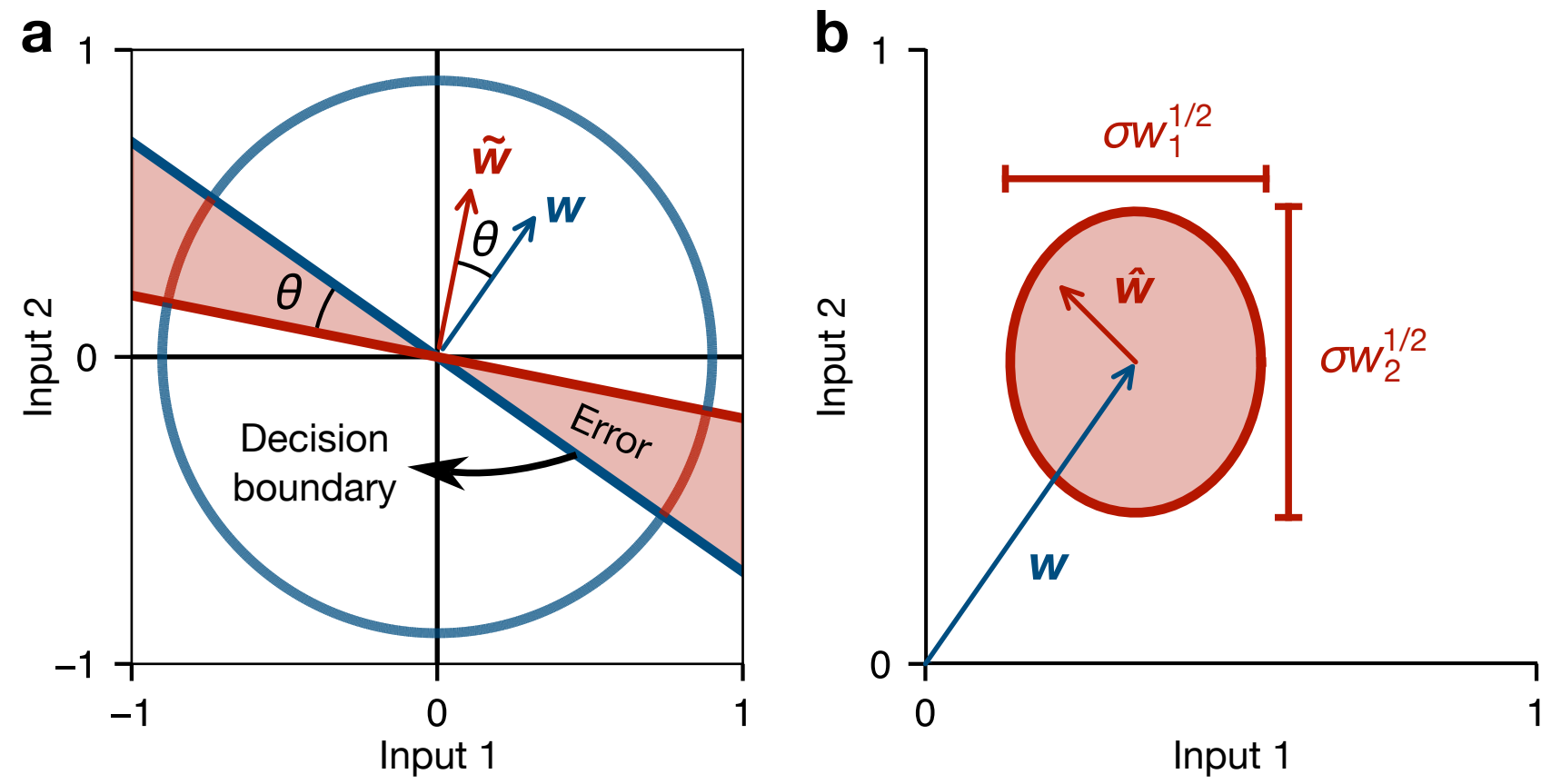

Fig. S1 | Geometric visualization of communication errors. **a**, Perturbations in the weights $\boldsymbol{w}$ shift the decision boundary of the neuron. When inputs are rotationally-symmetric, the probability of an error is proportional to the angular shift of the decision boundary. **b**, Input weights $\boldsymbol{w}$ define the distribution of weight perturbations $\hat{\boldsymbol{w}}$, which determines the distribution of the angle θ between $\boldsymbol{w}$ and the perturbed weights $\tilde{\boldsymbol{w}}$. The shaded red area shows a level set of the distribution of perturbed weights.

Letting $\langle w^2 \rangle = \frac{1}{n}\sum_{i=1}^{n} w_i^2$, we can write $\cos(\theta)$ as

$$\cos(\theta) = \frac{\boldsymbol{w}\cdot\tilde{\boldsymbol{w}}}{\|\boldsymbol{w}\|\|\tilde{\boldsymbol{w}}\|} = \frac{\langle w^2\rangle + \hat{A}}{\langle w^2\rangle^{1/2}(\langle w^2\rangle + 2\hat{A} + \hat{B})^{1/2}}, \tag{9}$$

where $\hat{A} = \frac{1}{n}\sum_{i=1}^{n} w_i \hat{w}_i$ and $\hat{B} = \frac{1}{n}\sum_{i=1}^{n} \hat{w}_i{}^2$. For large n, we expect the quantities $\hat{A}$ and $\hat{B}$ to concentrate around their average values (assuming $\langle w\rangle$ and $\langle w^2\rangle$ converge):

$$\mathbb{E}_{\tilde{\boldsymbol{w}}}(\hat{A}) = 0, \quad \mathbb{E}_{\tilde{\boldsymbol{w}}}(\hat{B}) = \sigma^2\langle w\rangle. \tag{10}$$

Indeed, all central moments of $\hat{A}$ and $\hat{B}$ are at most $\mathcal{O}(1/n)$. Therefore, expanding θ in terms of $\hat{A}$ and $\hat{B}$ around their averages yields a large-n expansion of the error rate:

$$\mathcal{E}(\boldsymbol{w}) = \frac{1}{\pi}\arccos\left(\frac{\langle w^2\rangle + \mathbb{E}_{\tilde{\boldsymbol{w}}}(\hat{A})}{\langle w^2\rangle^{1/2}(\langle w^2\rangle + 2\mathbb{E}_{\tilde{\boldsymbol{w}}}(\hat{A}) + \mathbb{E}_{\tilde{\boldsymbol{w}}}(\hat{B}))^{1/2}}\right) + \mathcal{O}\left(\frac{1}{n}\right). \tag{11}$$

Using the expectations in Eq. (10) and dropping higher-order terms, we obtain our analytical prediction of the error rate:

$$\mathcal{E}(\boldsymbol{w}) \approx \frac{1}{\pi}\arctan\left(\frac{\sigma}{r(\boldsymbol{w})}\right). \tag{12}$$

bioRxiv preprint doi: https://doi.org/10.64898/2026.10.21.760834; this version posted October 26, 2026. The copyright holder for this preprint (which was not certified by peer review) is the author/funder, who has granted bioRxiv a license to display the preprint in perpetuity. It is made available under a CC-BY 4.0 International license.

The analysis above gives an alternate intuition for what drives errors. Perturbations generate a distribution of weights centered around the unperturbed weights $\boldsymbol{w}$ (Fig. S1b). The perturbations $\hat{\boldsymbol{w}}$ are, in expectation, orthogonal to the original vector ($\mathbb{E}_{\tilde{\boldsymbol{w}}}(\hat{A}) = 0$). Thus, what determines the magnitude of θ is mostly the magnitude of perturbations in the orthogonal direction ($\mathbb{E}_{\tilde{\boldsymbol{w}}}(\hat{B}) = \sigma^2\langle w\rangle$). These intuitions about errors being related to the angles between decision boundaries apply roughly to our binary input setup. While the results for fixed perturbations are specific to rotationally-invariant distributions (like the Gaussian), we get the same quantitative behavior once we average over perturbations.

3. Sparse inputs and outputs

Our prediction of the error rate assumes that there is no bias in the local field and that inputs are zero-mean. This implies that the unperturbed neuron fires half the time, since the symmetry implies $\mathbb{P}\{z > 0\} = 1/2$. We can use our framework to explore how the error rate changes when we allow inputs and outputs to be sparse, and how the distribution of weights determines this behavior.

To relax the assumption of symmetric inputs, we now allow inputs to be drawn independently from a distribution with mean μ_x and variance σ_x^2. For example, this encompasses our original binary input setup with different probabilities of firing and not firing. We still assume that inputs are independently drawn. If the probability of staying silent is higher than the probability of firing, then $\mu_x < 0$. We also relax the assumption that there is no bias in the local field, which affects the firing probability of the postsynaptic neuron. We introduce a bias b to the local field:

$$z = \sum_i w_i x_i + b. \tag{13}$$

Decreasing the bias leads to a decrease in the firing rate of the neuron.

Allowing sparse inputs and outputs, we again make progress by studying the $n \to \infty$ limit. However, in this limit we must be careful about the scaling of b and μ_x. Rather than keeping μ_x and b fixed, we instead keep the probability that the neuron fires, which we call p_f, constant as the number of inputs n grows.

bioRxiv preprint doi: https://doi.org/10.64898/2026.10.21.760834; this version posted October 26, 2026. The copyright holder for this preprint (which was not certified by peer review) is the author/funder, who has granted bioRxiv a license to display the preprint in perpetuity. It is made available under a CC-BY 4.0 International license.

We begin our asymptotic analysis by studying the distribution of the unperturbed local field. It is useful to define the centered local field $z_c = \sum_i w_i(x_i - \mu_x)$ and characterize its asymptotic behavior. As we let n grow, we assume weights are drawn independently and that σ_x is held constant. The results, however, also hold if σ_x is allowed to vary but converges to an $\mathcal{O}(1)$ value. Under these assumptions, we can apply the central limit theorem to conclude that the centered local field is asymptotically Gaussian:

$$\frac{z_c}{\sqrt{n}} \xrightarrow{d} \mathcal{N}\left(0, \mathbb{E}_w(w^2)\sigma_x^2\right), \tag{14}$$

where $\xrightarrow{d}$ represents convergence in distribution. Note that this result holds regardless of the asymptotic behavior of μ_x and b.

Knowing the behavior of z_c allows us to study the distribution of z, even for finite n. For a fixed $\boldsymbol{w}$, note that the expected value of z, which we call μ_z, is $\mu_z = n\mu_x\langle w\rangle + b$. Therefore, we can decompose the local field as

$$z = \mu_z + z_c. \tag{15}$$

This decomposition allows us to characterize the firing rate p_f. The probability of firing is

$$p_f = \mathbb{P}\{z > 0\} = \mathbb{P}\left\{\frac{z_c}{n^{1/2}} > -\frac{\mu_z}{n^{1/2}}\right\}. \tag{16}$$

We can use the asymptotic behavior of $z_c/n^{1/2}$ to approximate the firing rate (Fig. S2a). For a given $\boldsymbol{w}$, we use $\mathbb{E}_w(w^2) \approx \langle w^2\rangle$ to obtain

$$p_f \approx \Phi\left(\frac{\mu_z}{n^{1/2}\langle w^2\rangle^{1/2}\sigma_x}\right), \tag{17}$$

where Φ is the CDF of the standard normal distribution. Note that to maintain a firing rate $p_f \neq 1/2$ that does not approach 0 or 1, we must have $\mu_z \propto n^{1/2}$. Thus, a sparse, but finite, firing rate can be achieved with $\mu_x \propto n^{-1/2}$ or $b \propto n^{1/2}$.

Having characterized the behavior of the unperturbed local field in terms of the firing rate, we can now study the effect of perturbations in sparse neurons. It is useful to work with the centered

bioRxiv preprint doi: https://doi.org/10.64898/2026.10.21.760834; this version posted October 26, 2026. The copyright holder for this preprint (which was not certified by peer review) is the author/funder, who has granted bioRxiv a license to display the preprint in perpetuity. It is made available under a CC-BY 4.0 International license.

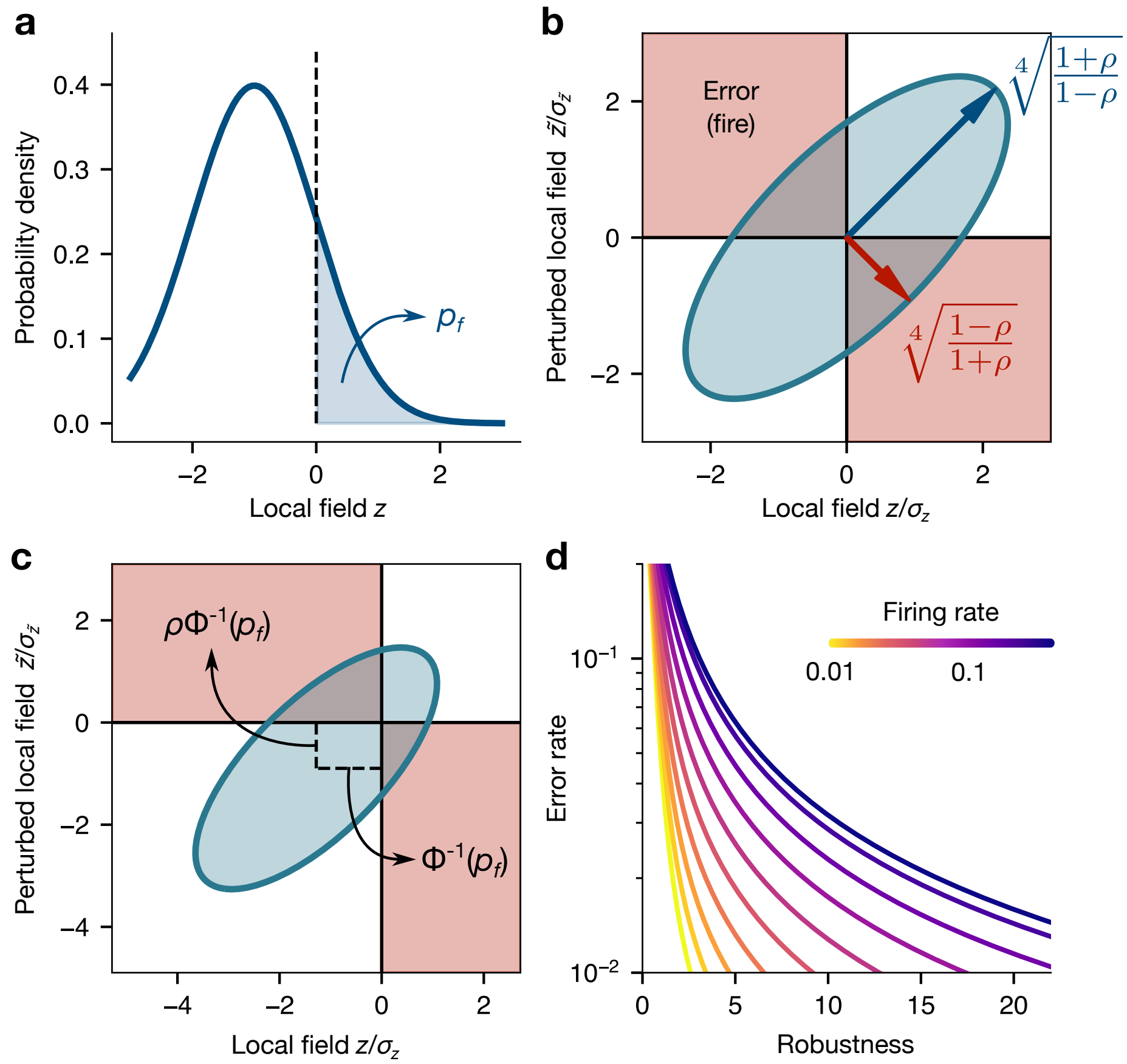

Fig. S2 | Geometric visualization of errors when inputs and outputs are sparse. a, Under non-symmetric inputs or non-zero bias, the local field distribution can be characterized asymptotically by the firing probability $p_f = \mathbb{P}\{z > 0\}$. **b**, Errors occur in the second and fourth quadrants in the space of local fields and perturbed local fields. For symmetric inputs and zero bias, the joint distribution of $(z/\sigma_z, \tilde{z}/\sigma_{\tilde{z}})$ is fully characterized by the correlation ρ between z and $\tilde{z}$. The shaded blue region represents a level set of the distribution. Arrows show the principal axes of the elliptic level set. **c**, When the unperturbed firing probability is $p_f \neq 1/2$, the distribution of $(z/\sigma_z, \tilde{z}/\sigma_{\tilde{z}})$ is shifted away from the origin. **d**, Error rate vs robustness for perturbation strength $\sigma = 1$ at varying firing rates p_f.

version of the perturbed local field: $\tilde{z}_c = \tilde{z} - \mu_z = z_c + \hat{z}$. We can use the same convergence result as in the main text for the joint distribution of $(z_c, \tilde{z}_c)$:

$$\frac{1}{\sqrt{n}}\begin{pmatrix} z_c \\ \tilde{z}_c \end{pmatrix} \xrightarrow{d} \mathcal{N}\left(\begin{pmatrix} 0 \\ 0 \end{pmatrix}, \sigma_x^2 \begin{pmatrix} \mathbb{E}_w(w^2) & \mathbb{E}_w(w^2) \\ \mathbb{E}_w(w^2) & \mathbb{E}_w(w^2) + \sigma^2(\mathbb{E}_x(x^2)/\sigma_x^2)\mathbb{E}_w(w) \end{pmatrix}\right). \tag{18}$$

For our analysis, in which the local field has non-zero mean, it is useful to state this result in terms

bioRxiv preprint doi: https://doi.org/10.64898/2026.10.21.760834; this version posted October 26, 2026. The copyright holder for this preprint (which was not certified by peer review) is the author/funder, who has granted bioRxiv a license to display the preprint in perpetuity. It is made available under a CC-BY 4.0 International license.

of the normalized version of the variables:

$$\begin{pmatrix} z_c/\sigma_z \\ \tilde{z}_c/\sigma_{\tilde{z}} \end{pmatrix} \xrightarrow{d} \mathcal{N}\left(\begin{pmatrix} 0 \\ 0 \end{pmatrix}, \begin{pmatrix} 1 & \rho \\ \rho & 1 \end{pmatrix} \right), \tag{19}$$

where the variances are

$$\sigma_z^2 = n\sigma_x^2 \mathbb{E}_w(w^2), \quad \sigma_{\tilde{z}}^2 = \sigma_z^2 + n\sigma_x^2\sigma^2(\mathbb{E}_x(x^2)/\sigma_x^2)\mathbb{E}_w(w) \tag{20}$$

and ρ is the correlation between z_c and $\tilde{z}_c$:

$$\rho = \left[1 + \sigma^2 \frac{\mathbb{E}_w(w)}{\mathbb{E}_w(w^2)} \frac{\mathbb{E}_x(x^2)}{\sigma_x^2}\right]^{-1/2}. \tag{21}$$

In $(z_c/\sigma_z, \tilde{z}_c/\sigma_{\tilde{z}})$ space, the correlation ρ sets the shape of the asymptotic joint distribution. In this space, the level sets of the joint distribution are ellipses whose major axis lies on the $y = x$ line. The correlation sets how much the ellipse is stretched along this axis, with a higher correlation leading to a more elongated ellipse. For $p_f = 1/2$ this elongation sets an intuitive picture for understanding the asymptotic role of the correlation in the error rate (Fig. S2b).

With our characterization of the asymptotic behavior of z_c and $\tilde{z}_c$, we can analyze the distribution of $(z, \tilde{z})$ to obtain the error rate. Note that we can (asymptotically) write the mean of z in terms of the firing probability p_f:

$$\mu_z = \sigma_z \Phi^{-1}(p_f). \tag{22}$$

Using this result, the means of our normalized local fields are

$$\mathbb{E}_{\boldsymbol{x}}\left(\frac{z}{\sigma_z}\right) = \Phi^{-1}(p_f), \quad \mathbb{E}_{\hat{\boldsymbol{w}},\boldsymbol{x}}\left(\frac{\tilde{z}}{\sigma_{\tilde{z}}}\right) = \frac{\sigma_z}{\sigma_{\tilde{z}}}\Phi^{-1}(p_f) = \rho\Phi^{-1}(p_f). \tag{23}$$

With this result, we see that the joint distribution of $(z/\sigma_z, \tilde{z}/\sigma_{\tilde{z}})$ is fully characterized by the correlation ρ and the firing rate p_f (Fig. S2c). Therefore, the asymptotic error rate is also a function of only ρ and p_f.

To compute the error rate $\mathcal{E}(\rho; p_f)$, we decompose the error probabilities as

$$\mathcal{E}(\rho; p_f) = \mathbb{P}\{z > 0, \tilde{z} < 0\} + \mathbb{P}\{z < 0, \tilde{z} > 0\}. \tag{24}$$

bioRxiv preprint doi: https://doi.org/10.64898/2026.10.21.760834; this version posted October 26, 2026. The copyright holder for this preprint (which was not certified by peer review) is the author/funder, who has granted bioRxiv a license to display the preprint in perpetuity. It is made available under a CC-BY 4.0 International license.

Note that

$$
\begin{aligned}
\mathbb{P}\{z > 0, \tilde{z} < 0\} &= \mathbb{P}\{z > 0\} - \mathbb{P}\{z > 0, \tilde{z} > 0\}, \\
\mathbb{P}\{z < 0, \tilde{z} > 0\} &= \mathbb{P}\{\tilde{z} > 0\} - \mathbb{P}\{z > 0, \tilde{z} > 0\}.
\end{aligned} \tag{25}
$$

Therefore, the error rate is

$$
\mathcal{E}(\rho; p_f) = \mathbb{P}\{z > 0\} + \mathbb{P}\{\tilde{z} > 0\} - 2\mathbb{P}\{z > 0, \tilde{z} > 0\}. \tag{26}
$$

Equivalently, in terms of normalized variables,

$$
\mathcal{E}(\rho; p_f) = \mathbb{P}\left\{\frac{z}{\sigma_z} > 0\right\} + \mathbb{P}\left\{\frac{\tilde{z}}{\sigma_{\tilde{z}}} > 0\right\} - 2\mathbb{P}\left\{\frac{z}{\sigma_z} > 0, \frac{\tilde{z}}{\sigma_{\tilde{z}}} > 0\right\}. \tag{27}
$$

Using our characterization of the joint distribution of the normalized local fields, these probabilities can be written explicitly:

$$
\mathcal{E}(\rho; p_f) = p_f + \Phi(\rho\Phi^{-1}(p_f)) - 2\Phi_2(\Phi^{-1}(p_f), \rho\Phi^{-1}(p_f); \rho), \tag{28}
$$

where Φ_2 is the bivariate standard normal CDF. As mentioned above, this error rate depends only on ρ and p_f. For finite n, we can approximate the correlation using robustness:

$$
\rho \approx \left[1 + \left(\frac{\sigma}{r(\boldsymbol{w})}\right)^2 \frac{\mathbb{E}_x(x^2)}{\sigma_x^2}\right]^{-1/2}. \tag{29}
$$

Thus, we have an analytical prediction of the error rate for sparsely-firing neurons that depends only on robustness and the firing rate. Specifically, the error rate is decreasing in robustness (as before) and increasing in the firing rate (Fig. S2d).

4. Correlated inputs

In this Section, we study the asymptotic behavior of the error rate when inputs are allowed to be correlated. We restrict ourselves to the case of $x_i \in \{-1, +1\}$ and $b = 0$. We also assume that $\mathbb{E}_x(x_i) = 0$, which implies that $\text{Var}(x_i) = 1$. For a fixed weight vector $\boldsymbol{w}$, the covariance matrix of the local field z and perturbed local field $\tilde{z}$ conditional on $\boldsymbol{w}$ is

$$
\Sigma_{\boldsymbol{w}} = \begin{pmatrix} \mathbb{E}_{\hat{\boldsymbol{w}},\boldsymbol{x}}(z^2) & \mathbb{E}_{\hat{\boldsymbol{w}},\boldsymbol{x}}(z\tilde{z}) \\ \mathbb{E}_{\hat{\boldsymbol{w}},\boldsymbol{x}}(\tilde{z}z) & \mathbb{E}_{\hat{\boldsymbol{w}},\boldsymbol{x}}(\tilde{z}^2) \end{pmatrix} = \begin{pmatrix} \sum_{i,j} w_i w_j c_{ij} & \sum_{i,j} w_i w_j c_{ij} \\ \sum_{i,j} w_i w_j c_{ij} & \sum_{i,j} w_i w_j c_{ij} + \sigma^2 \sum_i w_i \end{pmatrix}, \tag{30}
$$

bioRxiv preprint doi: https://doi.org/10.64898/2026.10.21.760834; this version posted October 26, 2026. The copyright holder for this preprint (which was not certified by peer review) is the author/funder, who has granted bioRxiv a license to display the preprint in perpetuity. It is made available under a CC-BY 4.0 International license.

where $c_{ij} = \text{Cov}(x_i, x_j)$. If we now assume that the weights $\boldsymbol{w}$ are drawn iid from a distribution $P(w)$, and independently from the inputs, the unconditional covariance matrix is

$$\mathbb{E}_{\boldsymbol{w}}(\Sigma_{\boldsymbol{w}}) = n \begin{pmatrix} \text{Var}(w) + q_n \mathbb{E}_w(w)^2 & \text{Var}(w) + q_n \mathbb{E}_w(w)^2 \\ \text{Var}(w) + q_n \mathbb{E}_w(w)^2 & \text{Var}(w) + q_n \mathbb{E}_w(w)^2 + \sigma^2 \mathbb{E}_w(w) \end{pmatrix}, \tag{31}$$

where $q_n = \frac{1}{n} \sum_{i,j} c_{ij}$. Thus, the correlation between z and $\tilde{z}$ at a finite number of inputs is

$$\rho_n = \left(1 + \frac{\sigma^2 \mathbb{E}_w(w)}{\text{Var}(w) + q_n \mathbb{E}_w(w)^2}\right)^{-1/2}. \tag{32}$$

We can write this correlation as $\rho_n = (1 + (\sigma / r_n^q)^2)^{-1/2}$, where we now use a modified version of robustness:

$$r_n^q = \sqrt{\frac{\text{Var}(w)}{\mathbb{E}_w(w)} + q_n \mathbb{E}_w(w)}. \tag{33}$$

When inputs are uncorrelated, we have $q_n = 1$ and we recover our previous result. Robustness is still monotonically increasing with variance, and is monotonically increasing in the off-diagonal correlations between inputs.

While we can intuitively characterize the correlation between z and $\tilde{z}$ when inputs are correlated, the independence condition needed to apply the central limit theorem is violated. To extend our covariance result to a prediction about the error rate, we need to establish weaker conditions for convergence to Gaussianity.

We first establish conditions under which $\frac{1}{\sqrt{n}} z$ converges to a Gaussian as $n \to \infty$. We split the local field into two contributions:

$$\frac{z}{\sqrt{n}} = \mathbb{E}_w(w) S_n + T_n, \quad S_n = \frac{1}{\sqrt{n}} \sum_{j=1}^{n} x_j, \quad T_n = \frac{1}{\sqrt{n}} \sum_{j=1}^{n} (w_j - \mathbb{E}_w(w)) x_j. \tag{34}$$

This decomposition allows us to break down our analysis into characterizing the asymptotic properties of two simpler objects. Intuitively, we can show that T_n converges to a Gaussian regardless of the distribution of $\boldsymbol{x}$. The difficulty is in obtaining sufficient conditions for S_n to also converge to a Gaussian.

bioRxiv preprint doi: https://doi.org/10.64898/2026.10.21.760834; this version posted October 26, 2026. The copyright holder for this preprint (which was not certified by peer review) is the author/funder, who has granted bioRxiv a license to display the preprint in perpetuity. It is made available under a CC-BY 4.0 International license.

To prove convergence we use a characteristic function approach. The characteristic function of the distribution of a single weight is

$$\varphi_w(t) = \mathbb{E}_w(e^{itw}). \tag{35}$$

It is useful to characterize the cumulant generating function $\log(\varphi_w(t))$. Expanding this function to second order yields

$$\log(\varphi_w(t)) = i\mathbb{E}_w(w)t - \frac{1}{2}\text{Var}(w)t^2 + t^2 h(t), \tag{36}$$

with $h \to 0$ as $t \to 0$.

Before studying the full characteristic function of $z/\sqrt{n}$, we compute the expectation of $e^{itz/\sqrt{n}}$ conditioned on an input realization $\boldsymbol{x}$. Since the weights are independent and the x_j are constant under the conditioning, the expectation factors as

$$\mathbb{E}_{\boldsymbol{w}}(e^{itz/\sqrt{n}}|\boldsymbol{x}) = \prod_{j=1}^{n} \mathbb{E}_w(e^{itx_j w/\sqrt{n}}|\boldsymbol{x}) = \exp\left[\sum_{j=1}^{n} \log\left(\varphi_w\left(\frac{tx_j}{\sqrt{n}}\right)\right)\right]. \tag{37}$$

Using our expansion of the cumulant generating function, we obtain

$$\mathbb{E}_{\boldsymbol{w}}(e^{itz/\sqrt{n}}|\boldsymbol{x}) = \exp\left[i\mathbb{E}_w(w)tS_n - \frac{1}{2}\text{Var}(w)t^2 + R_n(t,\boldsymbol{x})\right], \tag{38}$$

where the aggregate residual is

$$R_n(t,\boldsymbol{x}) = \frac{t^2}{n}\sum_{j=1}^{n} h\left(\frac{tx_j}{\sqrt{n}}\right). \tag{39}$$

Note that we can bound the size of this residual by

$$|R_n(t,\boldsymbol{x})| \le t^2\overline{h}\left(\frac{|t|}{\sqrt{n}}\right), \quad \overline{h}\left(\frac{|t|}{\sqrt{n}}\right) = \sup_{|u|\le|t|/\sqrt{n}} |h(u)|. \tag{40}$$

Importantly, note that the residual vanishes as $n \to \infty$ for all realizations $\boldsymbol{x}$ at any fixed t. Using our bound on the residual, we obtain the following for all inputs $\boldsymbol{x}$:

$$\begin{aligned}&\left|\mathbb{E}_{\boldsymbol{w}}(e^{itz/\sqrt{n}}|\boldsymbol{x}) - \exp\left[i\mathbb{E}_w(w)tS_n - \frac{1}{2}\text{Var}(w)t^2\right]\right| \\ &= \left|\exp\left[i\mathbb{E}_w(w)tS_n - \frac{1}{2}\text{Var}(w)t^2\right]\right|\left|1 - e^{R_n(t,\boldsymbol{x})}\right| \le e^{t^2\overline{h}(t/\sqrt{n})} - 1.\end{aligned} \tag{41}$$

bioRxiv preprint doi: https://doi.org/10.64898/2026.10.21.760834; this version posted October 26, 2026. The copyright holder for this preprint (which was not certified by peer review) is the author/funder, who has granted bioRxiv a license to display the preprint in perpetuity. It is made available under a CC-BY 4.0 International license.

Since the distance bound above is valid for all $\boldsymbol{x}$, it is also valid for the averages with respect to $\boldsymbol{x}$:

$$\left|\mathbb{E}_{\boldsymbol{w},\boldsymbol{x}}(e^{itz/\sqrt{n}}) - \mathbb{E}_{\boldsymbol{x}}\left(\exp\left[i\mathbb{E}_w(w)tS_n - \frac{1}{2}\text{Var}(w)t^2\right]\right)\right| \leq e^{t^2\overline{h}(t/\sqrt{n})} - 1. \tag{42}$$

Note that $\mathbb{E}_{\boldsymbol{w},\boldsymbol{x}}(e^{itz/\sqrt{n}})$ is the characteristic function of $z/\sqrt{n}$ and $\mathbb{E}_{\boldsymbol{x}}(e^{itS_n})$ is the characteristic function of S_n. Therefore, our bound reads

$$\left|\varphi_{z/\sqrt{n}}(t) - e^{-\text{Var}(w)t^2/2}\varphi_{S_n}(\mathbb{E}_w(w)t)\right| \leq e^{t^2\overline{h}(t/\sqrt{n})} - 1. \tag{43}$$

Up to this point we have made no assumptions on the joint distribution of $\boldsymbol{x}$, other than $x_i \in \{-1, +1\}$ and $\mathbb{E}_x(x_i) = 0$. To establish convergence to Gaussianity, we need the characteristic function to converge to asymptotically have the form $e^{i\mu t - \tau^2 t^2/2}$. This means that our modified central limit theorem requires that S_n converges to a Gaussian. We know $\mathbb{E}_{\boldsymbol{x}}(S_n) = 0$ and

$$\text{Var}(S_n) = \frac{1}{n}\sum_{j,k} c_{jk} = q_n. \tag{44}$$

Therefore, we can guarantee S_n converges to a Gaussian if $q_n \to q$ for some fixed q and

$$\left|\varphi_{S_n}(t) - e^{-qt^2/2}\right| = o(1), \tag{45}$$

where $o(1)$ means the value goes to 0 as $n \to \infty$. Under these conditions, we can use our previous distance bound to conclude that the characteristic function of $z/\sqrt{n}$ satisfies

$$\varphi_{z/\sqrt{n}}(t) \to e^{-[\text{Var}(w)+q\mathbb{E}_w(w)^2]t^2/2} \tag{46}$$

for all t. The convergence of the characteristic function implies convergence in distribution, meaning that

$$\frac{z}{\sqrt{n}} \xrightarrow{d} \mathcal{N}(0, \text{Var}(w) + q\mathbb{E}_w(w)^2). \tag{47}$$

What conditions are sufficient for S_n to converge to a Gaussian? A basic necessary condition is that q_n needs to remain bounded, which means that the average correlation $\frac{1}{n^2}\sum_{j,k} c_{jk}$ must go

bioRxiv preprint doi: https://doi.org/10.64898/2026.10.21.760834; this version posted October 26, 2026. The copyright holder for this preprint (which was not certified by peer review) is the author/funder, who has granted bioRxiv a license to display the preprint in perpetuity. It is made available under a CC-BY 4.0 International license.

to 0. However, this is not a sufficient condition. An intuitive sufficient condition can be formulated in terms of dependency graphs. In a dependency graph of random variables, any two disconnected sets of vertices correspond to mutually independent families of variables. Note that dependency graph need not be unique. Defining D_n as the maximum degree of a dependency graph of $\boldsymbol{x}$, a sufficient condition for Gaussian convergence is that $D_n = o(n^{1/4})$ (Theorem 4.3 in Ref. 66). Intuitively, this condition guarantees that there are no "global" couplings that can collectively perturb the distribution of z. For example, if the inputs were perfectly correlated, z would be composed of two Diracs. There also exist conditions on the (conditional) correlations between variables (Theorem 1 in Ref. 67), which are not as strict as independence but are significantly more complex to evaluate. These conditions encode the intuition that learning about one variable cannot reveal too much information about the rest of the variables.

We now return to the distribution of $(z/\sqrt{n}, \tilde{z}/\sqrt{n})$ and analyze its convergence. To extend our result to the multivariate case, we use the Cramér-Wold theorem,[68] which formulates sufficient conditions for multivariate convergence starting from single-variable convergence. For our application, to prove that $(z/\sqrt{n}, \tilde{z}/\sqrt{n})$ converges to a Gaussian with variances σ_1^2 and σ_2^2 and covariance C, it is sufficient to prove that the following holds for all $\alpha, \beta \in \mathbb{R}$:

$$\alpha\frac{z}{\sqrt{n}} + \beta\frac{\tilde{z}}{\sqrt{n}} \xrightarrow{d} \mathcal{N}(0, \alpha^2\sigma_1^2 + \beta^2\sigma_2^2 + 2\alpha\beta C). \tag{48}$$

We can decompose the relevant linear combination as

$$\alpha\frac{z}{\sqrt{n}} + \beta\frac{\tilde{z}}{\sqrt{n}} = \frac{1}{\sqrt{n}}\sum_{j=1}^{n}((\alpha+\beta)w_j + \beta\hat{w}_j)x_j. \tag{49}$$

Note that the random variables $(\alpha+\beta)w_j + \beta\hat{w}_j$ are iid, so we can reproduce the proof for convergence in distribution of z by using the replacement $w \to (\alpha+\beta)w + \beta\hat{w}$. We need the following statistics:

$$\begin{aligned}\mathbb{E}_{w,\hat{w}}((\alpha+\beta)w + \beta\hat{w}) &= (\alpha+\beta)\mathbb{E}_w(w),\\ \mathrm{Var}((\alpha+\beta)w + \beta\hat{w}) &= (\alpha+\beta)^2\mathrm{Var}(w) + \beta^2\sigma^2\mathbb{E}_w(w).\end{aligned} \tag{50}$$

bioRxiv preprint doi: https://doi.org/10.64898/2026.10.21.760834; this version posted October 26, 2026. The copyright holder for this preprint (which was not certified by peer review) is the author/funder, who has granted bioRxiv a license to display the preprint in perpetuity. It is made available under a CC-BY 4.0 International license.

Therefore, under the same assumptions that give convergence of z, we obtain

$$\alpha \frac{z}{\sqrt{n}} + \beta \frac{\tilde{z}}{\sqrt{n}} \xrightarrow{d} \mathcal{N}(0, (\alpha+\beta)^2(\text{Var}(w) + q\mathbb{E}_w(w)^2) + \beta^2\sigma^2\mathbb{E}_w(w)). \tag{51}$$

We conclude that the joint distribution satisfies the following convergence result:

$$\frac{1}{\sqrt{n}}\begin{pmatrix} z \\ \tilde{z} \end{pmatrix} \xrightarrow{d} \mathcal{N}\left(\begin{pmatrix} 0 \\ 0 \end{pmatrix}, \begin{pmatrix} \text{Var}(w) + \mathbb{E}_w(w)^2 q & \text{Var}(w) + \mathbb{E}_w(w)^2 q \\ \text{Var}(w) + \mathbb{E}_w(w)^2 q & \text{Var}(w) + q\mathbb{E}_w(w)^2 + \sigma^2\mathbb{E}_w(w) \end{pmatrix}\right). \tag{52}$$

With this result, we recover our error rate with a modified robustness metric:

$$\mathcal{E} = \frac{1}{\pi}\arctan\left(\frac{\sigma}{r^q}\right), \quad r^q = \sqrt{\frac{\text{Var}(w)}{\mathbb{E}_w(w)} + q\mathbb{E}_w(w)}. \tag{53}$$

5. Other noise structures

In the main text, we focused on errors induced by additive noise at the synapse level. However, our framework allows for a more general weight perturbations. We still assume that the perturbations $\hat{w}_i$ satisfy $\mathbb{E}_{\hat{w}}(\hat{w}_i) = 0$ and are independently drawn. However, we now assume that their variance has a general structure,

$$\text{Var}(\hat{w}_i) = \sigma^2 v(w_i), \tag{54}$$

where v is an arbitrary function of the weights. The only condition we impose on v is that it does not make the variance of $\tilde{z}$ grow unbounded. We maintain the noise strength parameter σ for expositional clarity. The joint distribution of $(z, \tilde{z})$ now satisfies

$$\frac{1}{\sqrt{n}}\begin{pmatrix} z \\ \tilde{z} \end{pmatrix} \xrightarrow{d} \mathcal{N}\left(\begin{pmatrix} 0 \\ 0 \end{pmatrix}, \sigma_x^2\begin{pmatrix} \mathbb{E}_w(w^2) & \mathbb{E}_w(w^2) \\ \mathbb{E}_w(w^2) & \mathbb{E}_w(w^2) + \sigma^2\mathbb{E}_w(v(w)) \end{pmatrix}\right). \tag{55}$$

The computation of the error rate then follows as in the main text. This implies that, for fixed $\boldsymbol{w}$, we can make a prediction of the error rate $\mathcal{E}_v(\boldsymbol{w})$ associated to this noise structure:

$$\mathcal{E}_v(\boldsymbol{w}) \approx \frac{1}{\pi}\arccos\left(\left[1 + \left(\frac{\sigma}{r_v(\boldsymbol{w})}\right)^2\right]^{-1/2}\right), \tag{56}$$

bioRxiv preprint doi: https://doi.org/10.64898/2026.10.21.760834; this version posted October 26, 2026. The copyright holder for this preprint (which was not certified by peer review) is the author/funder, who has granted bioRxiv a license to display the preprint in perpetuity. It is made available under a CC-BY 4.0 International license.

where robustness now has the modified form

$$r_v(\boldsymbol{w}) = \sqrt{\frac{\langle w^2 \rangle}{\langle v(w) \rangle}}. \tag{57}$$

Therefore, the simplicity of our robustness prediction is not tied to our particular choice of noise structure; the error rate still depends on the weight only via a simple statistic. The noise structure only determines the specific form that robustness takes.

Consider noise of the form

$$v(w) = w^\eta, \tag{58}$$

which produces robustness,

$$r_\eta(\boldsymbol{w}) = \sqrt{\frac{\langle w^2 \rangle}{\langle w^\eta \rangle}}. \tag{59}$$

Note that the additive noise we consider in the main text corresponds to $\eta = 1$. This characterization allows for two additional important classes of noise. For $\eta = 0$, we obtain perturbations that have the same strength regardless of the weight. This constant noise could arise from processes that affect communication at the connection level, such as fluctuations at the level of entire presynaptic neurons. In this scenario, robustness monotonically increases with both the average connection strength $\langle w \rangle$ and variance $\mathrm{Var}(w)$. Another case of interest is $\eta = 2$, which corresponds to multiplicative noise. Multiplicative noise arises when there is a common noise source shared by all synapses, which is then amplified by each additional synapse. In this scenario, the robustness becomes independent of the connection strengths.

We can study which distributions of weights give a higher or lower robustness at a given η. For this, we fix the average weight $\langle w \rangle$ and ask which distributions have the highest or lowest robustness. For $\eta = 1$, we showed that the weight distribution with the lowest robustness is the uniform distribution, in which $w_i = \langle w \rangle$. In fact, this is true for all sub-multiplicative noise, $\eta \in [0, 2]$. To see this, note that by Jensen's inequality,

$$\langle w^\eta \rangle = \langle (w^2)^{\eta/2} \rangle \leq \langle w^2 \rangle^{\eta/2}. \tag{60}$$

bioRxiv preprint doi: https://doi.org/10.64898/2026.10.21.760834; this version posted October 26, 2026. The copyright holder for this preprint (which was not certified by peer review) is the author/funder, who has granted bioRxiv a license to display the preprint in perpetuity. It is made available under a CC-BY 4.0 International license.

Using this inequality and $\langle w^2 \rangle \geq \langle w \rangle^2$ (also by Jensen's inequality), we see that robustness satisfies

$$r_\eta(\boldsymbol{w}) = \sqrt{\frac{\langle w^2 \rangle}{\langle w^\eta \rangle}} \geq \sqrt{\langle w^2 \rangle^{1-\eta/2}} \geq \sqrt{\langle w \rangle^{2-\eta}} = r_\eta^{\text{uni}}(\boldsymbol{w}), \tag{61}$$

where $r_\eta^{\text{uni}}(\boldsymbol{w})$ is the robustness value of the uniform weight vector. We can also characterize the weights that maximize robustness. Assuming the weights are non-negative, the largest possible value of a given w_i is $n\langle w \rangle$. With this, we can obtain a lower bound for $\langle w^\eta \rangle$:

$$\langle w^\eta \rangle = \langle w^2 w^{\eta-2} \rangle \geq \langle w^2 (n\langle w \rangle)^{\eta-2} \rangle = n^{\eta-2} \langle w \rangle^{\eta-2} \langle w^2 \rangle. \tag{62}$$

Thus, we obtain an upper bound for robustness:

$$r_\eta(\boldsymbol{w}) \leq \sqrt{n^{2-\eta} \langle w \rangle^{2-\eta}}. \tag{63}$$

This bound can be achieved by the maximally-sparse weight vector: $\boldsymbol{w} = (n\langle w \rangle, 0, \ldots, 0)$ or any of its permutations. Calling the robustness of the single-entry weight vector $r_\eta^{\text{single}}(\boldsymbol{w})$, we see that, in general,

$$r_\eta^{\text{uni}}(\boldsymbol{w}) \leq r_\eta(\boldsymbol{w}) \leq r_\eta^{\text{single}}(\boldsymbol{w}) \quad (0 \leq \eta \leq 2). \tag{64}$$

For larger values of η, the relevant inequalities flip, giving the reverse order:

$$r_\eta^{\text{single}}(\boldsymbol{w}) \leq r_\eta(\boldsymbol{w}) \leq r_\eta^{\text{uni}}(\boldsymbol{w}) \quad (\eta \geq 2). \tag{65}$$

Thus, for sub-multiplicative noise $\eta \in [0, 2]$, high robustness requires the distribution of weights to be heavy-tailed. This is because distributions with heavier tails create wider distributions of local fields without producing a comparable effect in the distribution of the local field perturbation $\hat{z}$ (Fig. 2c in the main text). In contrast, when noise is super-multiplicative ($\eta > 2$), widening the distribution of the local field requires a super-linear widening of the local field perturbation, inducing more errors. Therefore, in this case it is beneficial to have the lowest possible spread in connection strengths, corresponding to the uniform distribution.

6. Communication with continuous output

bioRxiv preprint doi: https://doi.org/10.64898/2026.10.21.760834; this version posted October 26, 2026. The copyright holder for this preprint (which was not certified by peer review) is the author/funder, who has granted bioRxiv a license to display the preprint in perpetuity. It is made available under a CC-BY 4.0 International license.

Our main analysis focused on binary inputs, motivated by spiking neurons. However, we can extend our framework to analyze how perturbations induce errors in graded responses. As a simple example, consider neurons with a sigmoidal response to the local field. Motivated by our symmetric binary convention ($+1$ for firing and -1 for silence), we take the output to be

$$y(z;T) = \tanh\left(\frac{z}{n^{1/2}T}\right), \tag{66}$$

where T is a temperature parameter that modulates the size of the region in z space in which intermediate values of y are possible. This is because for $|z|/\sqrt{n} \gg T$ the outputs are essentially binary. The $\sqrt{n}$ scaling is introduced to avoid trivial behaviors in the model in the large n limit.

To quantitatively study the effects of perturbations in a continuous output setting, we need to generalize the concept of error rate used for binary neurons. We study a simple quadratic loss, where the pointwise loss obtained from a particular realization of an unperturbed output y and a perturbed one $\tilde{y}$ is

$$\ell(y,\tilde{y}) = \frac{1}{4}(y-\tilde{y})^2. \tag{67}$$

While we focus on this particular form of loss, our analysis would remain qualitatively the same for any smooth loss function. Note that when $T = 0$, this quadratic loss coincides with the misclassification loss $\ell(y,\tilde{y}) = 1(y \neq \tilde{y})$, where $1(\cdot)$ is the indicator function. The performance of a neuron is quantified by its average loss (or simply loss)

$$\mathcal{L}(\boldsymbol{w};T) = \mathbb{E}_{\hat{\boldsymbol{w}},\boldsymbol{x}}[\ell(y(z;T), y(\tilde{z};T))]. \tag{68}$$

As the temperature parameter approaches 0 the loss simply becomes our original error rate:

$$\lim_{T\to 0} \mathcal{L}(\boldsymbol{w};T) = \mathcal{E}(\boldsymbol{w}). \tag{69}$$

The sigmoidal activation naturally gives rise to two regimes. As mentioned above, $|z|/\sqrt{n} \gg T$ corresponds to a saturated regime, in which the output is essentially binary, and small changes in the local field do not affect the output. By contrast, when $|z|/\sqrt{n} \ll T$ the output is approximately a linear function of the local field, meaning that small changes in the local field have proportional

bioRxiv preprint doi: https://doi.org/10.64898/2026.10.21.760834; this version posted October 26, 2026. The copyright holder for this preprint (which was not certified by peer review) is the author/funder, who has granted bioRxiv a license to display the preprint in perpetuity. It is made available under a CC-BY 4.0 International license.

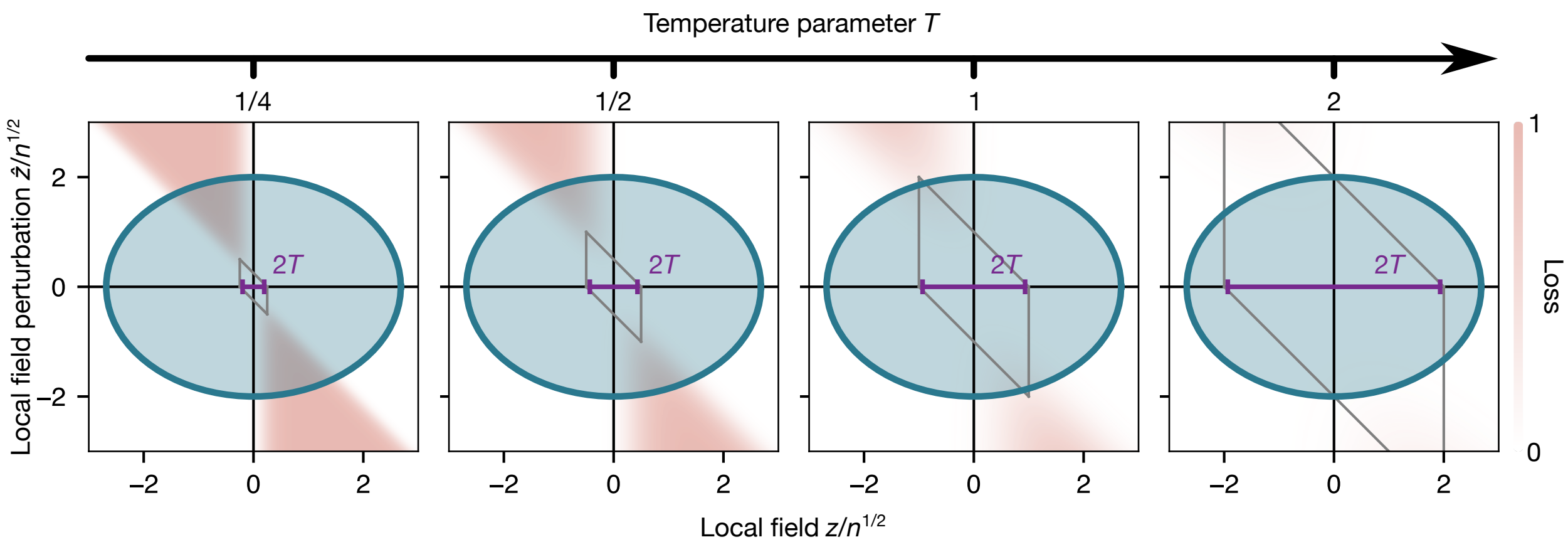

Fig. S3 | Visualization of loss under continuous outputs. The joint distribution of the local field and the local field perturbation (level set shown in blue) determine the loss. The temperature parameter T sets the size of the linear region (gray parallelogram). For very low temperatures, the linear region is much smaller than the scale of the distribution, and the loss behaves as the binary loss in the main text. For very large temperatures, the bulk of the distribution is in the linear region, and the loss becomes proportional to $\hat{z}^2/nT^2$.

effects on the output. This behavior translates to a separation of $(z, \hat{z})$ space into regions where outputs are approximately linear or binary. For a given $\boldsymbol{w}$, the qualitative behavior of the loss depends on how much the distribution of $(z, \hat{z})$ overlaps with the linear region (Fig. S3). For large temperatures, the bulk of this distribution is in the linear region, so the loss takes the form

$$\mathcal{L}(\boldsymbol{w}; T) = \frac{\mathbb{E}_{\boldsymbol{w},\boldsymbol{x}}[(z - \tilde{z})^2]}{4nT^2} + \mathcal{O}\left(\frac{1}{T^3}\right). \tag{70}$$

Taking the expectation, we get

$$\mathcal{L}(\boldsymbol{w}; T) \approx \frac{\sigma^2 \langle w \rangle}{4T^2} \quad (T \gg \langle w^2 \rangle^{1/2}). \tag{71}$$

This result is in striking contrast to the loss at small T. In this linear regime, there is no benefit to having a large variance in weights: all weights $\boldsymbol{w}$ with the same $\langle w \rangle$ have approximately the same error. This is because widening the distribution of local fields no longer has the sharp effect of changing outcomes from an "error" region to a "correct" region. Instead, the linearity of the output means that for a fixed perturbation, changing the local field does not affect the loss. As the temperature parameter decreases, nonlinear effects become important and the advantages of

bioRxiv preprint doi: https://doi.org/10.64898/2026.10.21.760834; this version posted October 26, 2026. The copyright holder for this preprint (which was not certified by peer review) is the author/funder, who has granted bioRxiv a license to display the preprint in perpetuity. It is made available under a CC-BY 4.0 International license.

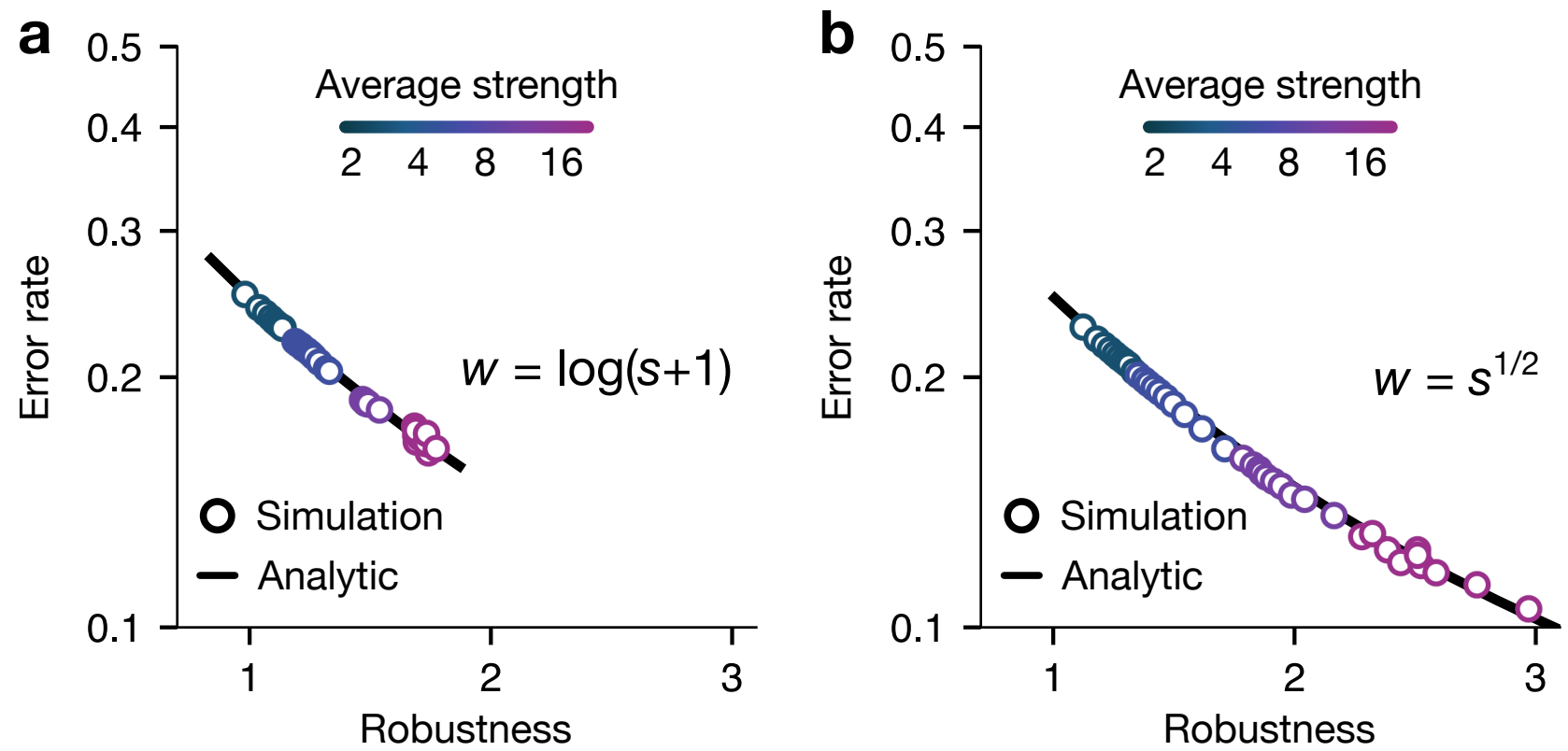

Fig. S4 | Error rates are determined by robustness in alternative definitions of connection strengths. a, Error rates versus robustness for neurons in the fruit fly brain[7,8] with at least ten incoming connections using connection strengths $w = \log(s + 1)$. Neurons were grouped into the same groups as Fig. 3 of the main text. The median simulated error rate and median robustness of each group are shown as scatter points. The solid line represents our analytical prediction. **b**, Error rates versus robustness using connection strengths $w = s^{1/2}$. For both scenarios, the perturbation strength is $\sigma = 1$.

having a wide distribution of weights become stronger. This result shows that the advantages of heavy-tailed distributions of connection strength are inherently tied to the nonlinearity of neuron responses.

7. Alternative definitions of connection strengths

In the main text, we used two different definitions of connection strength: synapse count for invertebrates and contact area for mouse retina. However, our framework allows for more general definitions of connection strengths. To illustrate this generality, we study errors for connection strengths of the form $w = f(s)$, where s is the number of synapses in a connection. Specifically, we consider $w = \log(s + 1)$ and $w = \sqrt{s}$. We find that our prediction for the error rate is in excellent agreement with simulations and that the specific choice of f only changes the distribution of robustness, but not the accuracy of our analytic prediction (Fig. S4). In simulations with $w = \log(s + 1)$, the predicted error rate is within 0.004 of the simulated one on average, compared to an average simulated error rate of 0.221. For $w = \sqrt{s}$, predicted error rate is within 0.004 of the simulated one on average, compared to an average simulated error rate of 0.197.

bioRxiv preprint doi: https://doi.org/10.64898/2026.10.21.760834; this version posted October 26, 2026. The copyright holder for this preprint (which was not certified by peer review) is the author/funder, who has granted bioRxiv a license to display the preprint in perpetuity. It is made available under a CC-BY 4.0 International license.

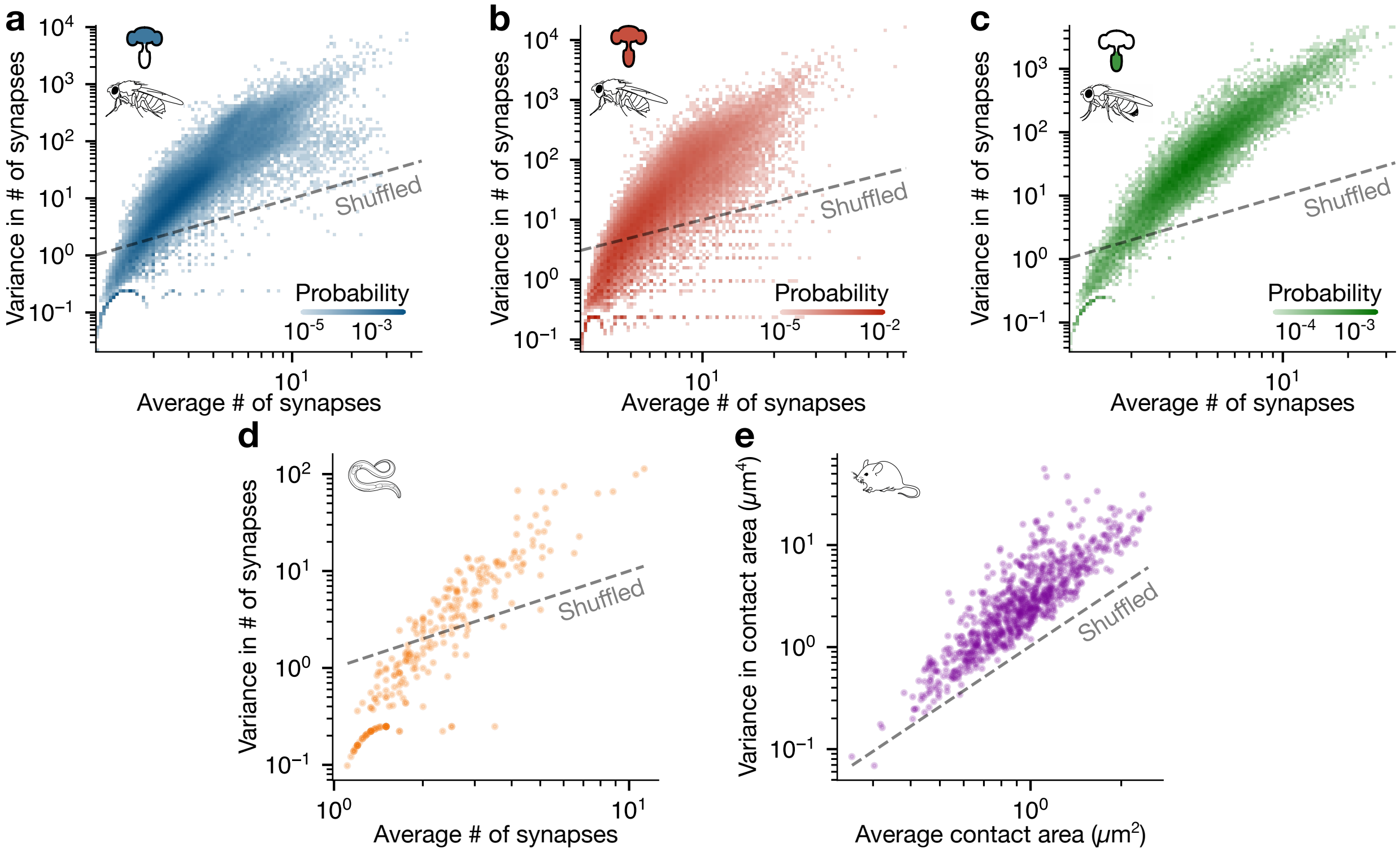

Fig. S5 | Connection strength statistics across species. a-d, Average number of synapses and variance in the number of synapses of the incoming connections of neurons in the female fruit fly brain (**a**),[7,8] the combined brain and nerve cord of a *Drosophila* female (**b**),[9] the *Drosophila* male nerve cord (**c**),[10] and the nematode *C. elegans* (**d**).[3,4] **e**, Average contact area and variance in the contact area of incoming connections of neurons in the inner plexiform layer of the mouse retina.[5] Dashed lines represent the variance-mean relationship after connections are shuffled ($\mathrm{Var}(w) = \langle w \rangle$ for invertebrates, $\mathrm{Var}(w) = \langle w \rangle^2 (k_{\mathrm{in}} - 1)/(k_{\mathrm{in}} + 1) \approx \langle w \rangle^2$ for the mouse retina).

We expect our error rate prediction to remain accurate for other choices of f. For our results to remain valid, the conditions for the convergence of the $(z, \hat{z})$ distribution must still hold. The central concern is that the distribution of w becomes too wide and large weights dominate the output. Formalizing this intuition, we can show that any f that reduces the spread in the weights guarantees convergence. For example, this is possible if f satisfies $|f'(s)| \leq 1$, as in the cases studied above.

8. Distribution of connection strength statistics

bioRxiv preprint doi: https://doi.org/10.64898/2026.10.21.760834; this version posted October 26, 2026. The copyright holder for this preprint (which was not certified by peer review) is the author/funder, who has granted bioRxiv a license to display the preprint in perpetuity. It is made available under a CC-BY 4.0 International license.

As shown in the main text, real neurons are consistently more robust than neurons with random synaptic connectivity. The robustness of a neuron is determined by the statistics of its incoming connection strengths. Thus, by analyzing the distribution of incoming statistics across neurons, we can obtain a more detailed picture of what drives their high robustness. We see that the joint distribution of means and variances in incoming connection strengths is consistently concentrated along a one-dimensional relationship across organisms, such that a high average connection strength is accompanied by a high variance (Fig. S5). This concentrated distribution of statistics is consistently above the $\mathrm{Var}(w) = \langle w \rangle$ benchmark, which corresponds to shuffling units of connection strength among connections. This concentration above the shuffled benchmark results in high robustness for most neurons.

9. Shuffling synapses in thresholded connectomes

In the connectome of the combined brain and nerve cord of a *Drosophila* female,[9] connections are thresholded, such that only connections with at least three synapses are reported. Thresholding introduces additional choices on how to construct null neurons for our analysis in Fig. 4 of the main text. There, we construct shuffled neurons by first placing three synapses into each incoming connection and then placing synapses uniformly at random until the total number of incoming synapses matches that of the real neuron. In this Section, we propose two alternative shuffling schemes and compare the robustness of the resulting neurons against the robustness of the real neurons.

In the first alternative scheme, synapses are shuffled in groups. Intuitively, we can interpret the threshold as imposing a resolution on synapse counts. We can account for this imposed resolution by placing synapses in groups of three uniformly at random, until the number of synapses matches the total number of the original neuron. The second alternative is to simply use the same scheme as the other invertebrate connectomes: assigning synapses to incoming connections uniformly at random until the total synaptic count matches the original neuron.

The top row of Fig. S6 shows the robustness of neurons before and after shuffling synapses

bioRxiv preprint doi: https://doi.org/10.64898/2026.10.21.760834; this version posted October 26, 2026. The copyright holder for this preprint (which was not certified by peer review) is the author/funder, who has granted bioRxiv a license to display the preprint in perpetuity. It is made available under a CC-BY 4.0 International license.

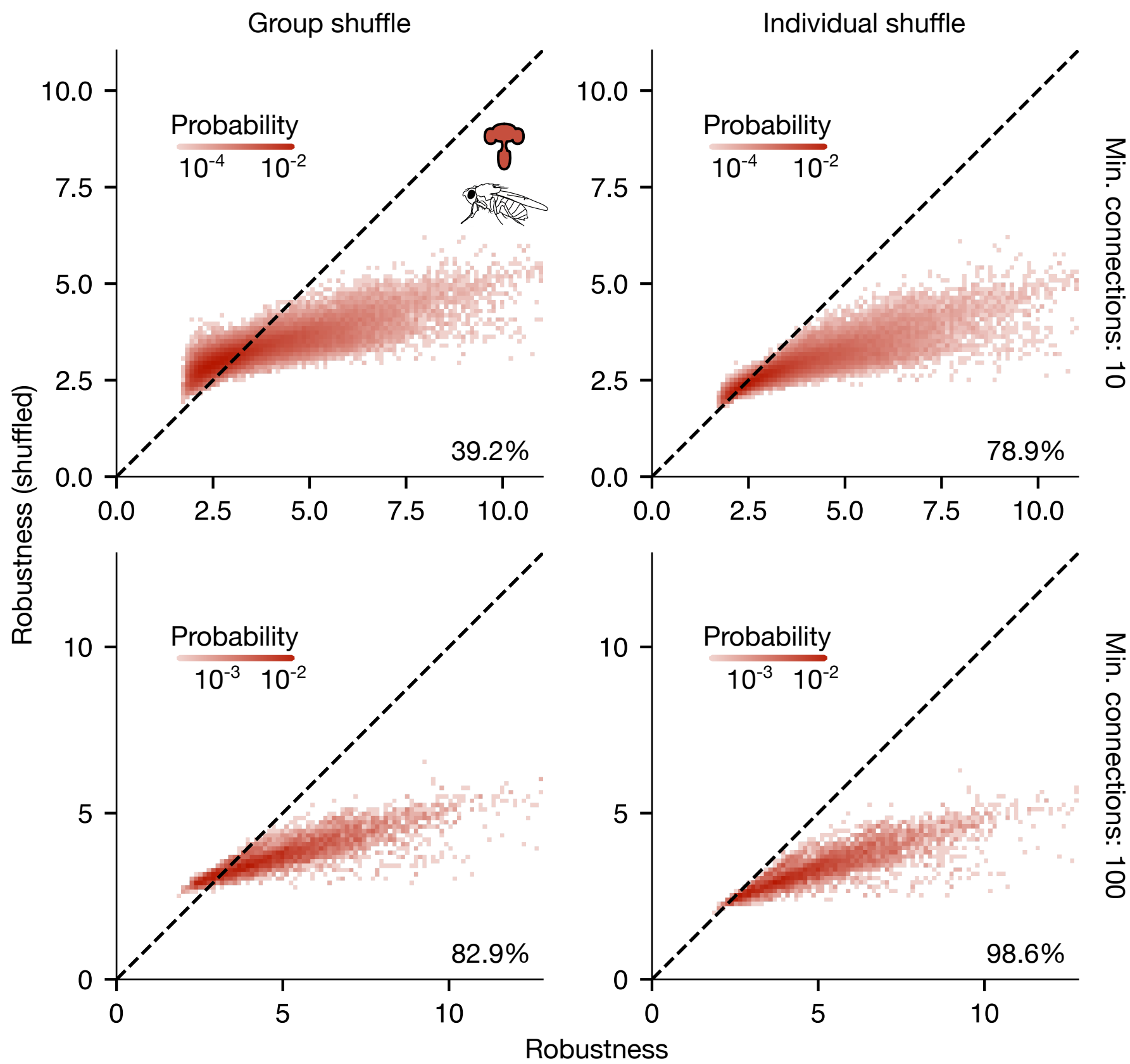

Fig. S6 | Robustness under different thresholding choices. Robustness of neurons in the fruit fly brain and nerve cord[9] before and after shuffling synapses among incoming connections. *Left column*: Synapses are placed into connections in groups of three. *Right column*: Synapses placed into connections individually. *Top row*: Comparison for neurons with at least ten incoming connections, as in the main text. *Bottom row*: Comparison for neurons with at least 100 incoming connections.

using the above schemes. We see that the fraction of neurons whose robustness is higher than their shuffled counterparts is lower than in Fig. 4 of the main text. This is because these alternative shuffling procedures can leave connections with zero synapses, which allows the distribution of connection strengths to have a higher variance and, hence, higher robustness. Intuitively, this is more important in neurons that have connections with low synaptic counts. This explains why only low-robustness neurons increase in robustness when shuffled.

To test whether the low fraction of high-robustness neurons is explained by neurons with few connections, we restrict our analysis to neurons with at least 100 incoming connections (Fig. S6,

bioRxiv preprint doi: https://doi.org/10.64898/2026.10.21.760834; this version posted October 26, 2026. The copyright holder for this preprint (which was not certified by peer review) is the author/funder, who has granted bioRxiv a license to display the preprint in perpetuity. It is made available under a CC-BY 4.0 International license.

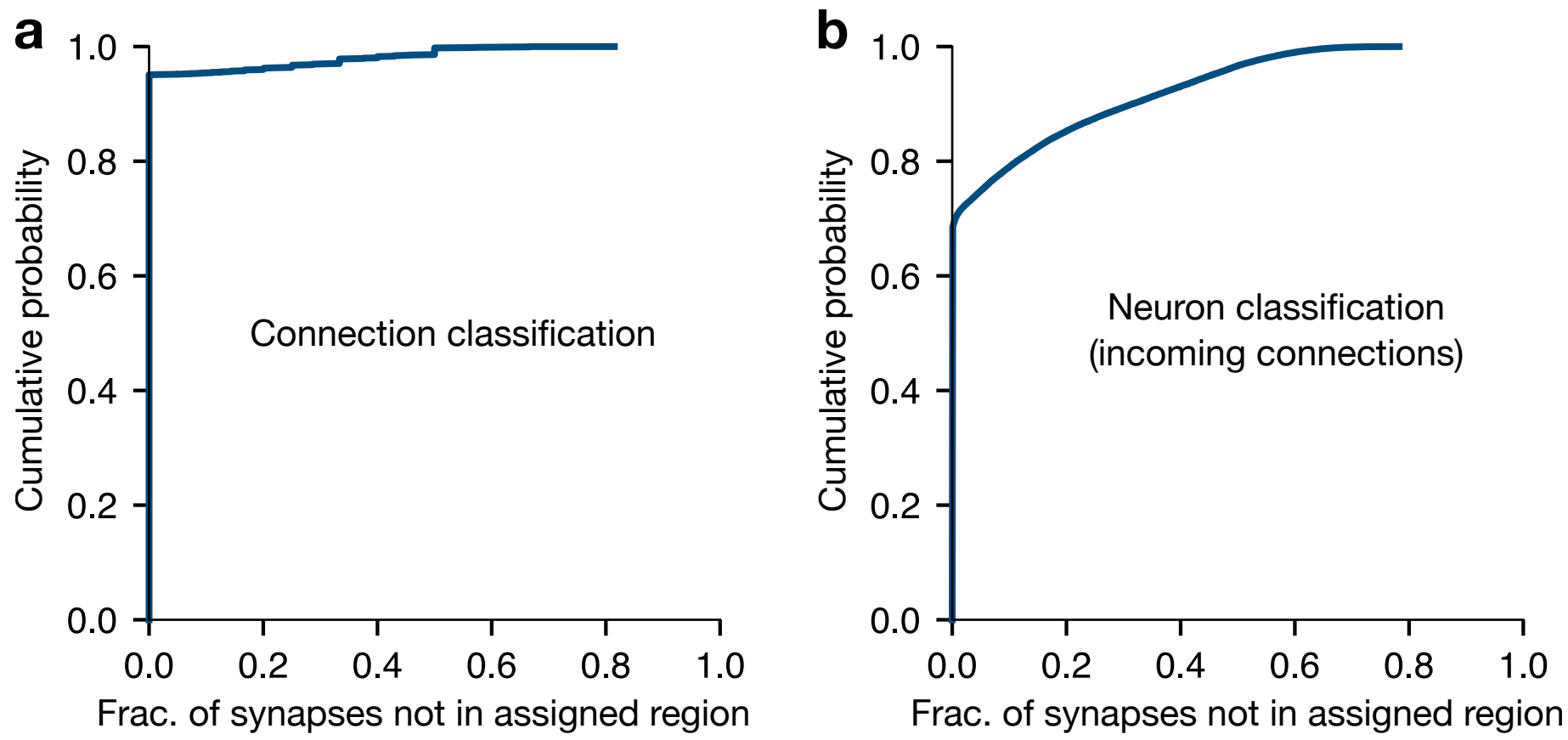

Fig. S7 | Synapse composition in plurality-classified connections and neurons. a, Distribution of the fraction of synapses in each connection that fall outside the connection's assigned brain region across the adult female fruit fly brain.[7,8] **b**, Distribution of the fraction of synapses in each neuron's incoming connections that fall outside the neuron's assigned brain region. Neurons were classified into brain regions using plurality of their incoming synapses.

bottom row). With this restriction, the low-robustness neurons driving this result are removed, such that the majority of the remaining neurons have higher robustness than their shuffled counterparts.

10. Plurality classification of connections and neurons

Our analysis of synaptic connectivity by brain region in the adult female fruit fly uses a classification of connections and neurons into specific brain regions. Ref. 7, 8 classifies individual synapses into neuropils, and each neuropil belongs to one brain region. We classify connections into brain regions by plurality of their synapses: each connection is assigned the region which contains the largest number of its synapses. Similarly, we assign neurons into brain regions by plurality of their incoming synapses. To quantify how much information is lost in this coarse-graining, we can study the distribution of the fraction of synapses that are in connections or neurons assigned to different regions (Fig. S7). We find that around 95% of connections have all synapses in a single region, and around 70% of neurons have all synapses from their incoming connections in a single region.
